# Biodegradable electrohydraulic actuators for sustainable soft robots

**DOI:** 10.1126/sciadv.adf5551

**Published:** 2023-03-22

**Authors:** Ellen H. Rumley, David Preninger, Alona Shagan Shomron, Philipp Rothemund, Florian Hartmann, Melanie Baumgartner, Nicholas Kellaris, Andreas Stojanovic, Zachary Yoder, Benjamin Karrer, Christoph Keplinger, Martin Kaltenbrunner

**Affiliations:** ^1^ Robotic Materials Department, Max Planck Institute for Intelligent Systems, Stuttgart, Germany.; ^2^ Paul M. Rady Department of Mechanical Engineering, University of Colorado, Boulder, CO, USA.; ^3^ Division of Soft Matter Physics, Institute for Experimental Physics, Johannes Kepler University, Linz, Austria.; ^4^ Soft Materials Lab, Linz Institute of Technology, Johannes Kepler University, Linz, Austria.; ^5^ Materials Science and Engineering Program, University of Colorado, Boulder, CO, USA.

## Abstract

Combating environmental pollution demands a focus on sustainability, in particular from rapidly advancing technologies that are poised to be ubiquitous in modern societies. Among these, soft robotics promises to replace conventional rigid machines for applications requiring adaptability and dexterity. For key components of soft robots, such as soft actuators, it is thus important to explore sustainable options like bioderived and biodegradable materials. We introduce systematically determined compatible materials systems for the creation of fully biodegradable, high-performance electrohydraulic soft actuators, based on various biodegradable polymer films, ester-based liquid dielectric, and NaCl-infused gelatin hydrogel. We demonstrate that these biodegradable actuators reliably operate up to high electric fields of 200 V/μm, show performance comparable to nonbiodegradable counterparts, and survive more than 100,000 actuation cycles. Furthermore, we build a robotic gripper based on biodegradable soft actuators that is readily compatible with commercial robot arms, encouraging wider use of biodegradable materials systems in soft robotics.

## Graphical abstract

**Figure:**
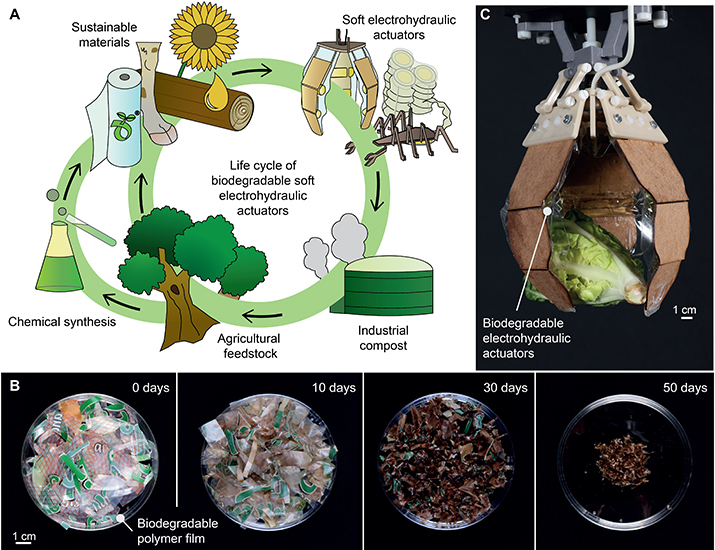

## Teaser

A biodegradable materials system that enables the development of high-performance yet sustainable soft electrostatic actuators.

## INTRODUCTION

Over the last decades, industries and consumers alike have come to rely on robots for performing tasks efficiently and reliably. Along with increased use of robotics, however, follows technological waste and unsustainable disposal practices; electronics generated 53.6 million metric tons of unrecycled waste in 2019 alone, a number expected to grow annually by nearly 2 million (*1*). The trend of accumulating waste raises a multitude of environmental and health concerns (*2*–*6*) and must be addressed by developers of advancing technologies such as robotics. On top of promoting a clean environment, they are also encouraged to use sustainable materials due to stricter regulations on waste production and raw material consumption in coming years (*7*) as well as the pressure to meet increasing consumer demands for green technology (*8*).

Sustainable material options consist of recyclables, renewables, and biodegradable materials (*9*). The latter category, particularly plant and animal products, offers a wide selection of mechanical properties such as tunable elasticity, stiffness, and yield strength. These material qualities are especially attractive for use in soft robotics, an emerging field that aims to use compliant structures to endow robots and machines with levels of dexterity and adaptability currently only seen in biological organisms (*10*–*14*). Just as biological muscles account for the majority of mass of the human body (*15*), soft actuators are equally integral parts of all soft robotic devices. This therefore warrants a pronounced focus on the development of sustainable soft actuator technologies and, more generally, for researchers to embrace sustainability as a key design goal for soft robotic devices.

Soft actuators are a subject of intense research and have been designed to respond to a range of stimuli, such as pressure, heat, chemical reactions, and magnetic or electric fields (*16*–*20*). Previous works have successfully leveraged the mechanical properties of biodegradable materials and implemented them into several unique soft actuator designs: Pneumatically driven actuators can consist of fully biodegradable components, using materials like biogels, cotton fibers, self-healing proteins, or seed-germinating foams (*21*–*24*) as mediums that enable large deformations in response to pressure; some thermally driven actuators can even harness the bursting of popcorn kernels to actuate (*25*).

Among the different types of soft actuators, soft electrostatic actuators that harness liquid dielectrics are particularly promising, achieving high actuation speeds, portability, energy efficiency, versatility, and silent operation (*26*–*35*); however, fully biodegradable materials systems for such actuators have yet to be identified. In their essence, they consist of solid polymer films, liquid dielectrics, and flexible electrodes; electric fields are used to generate Maxwell stresses that redistribute liquid dielectrics within soft actuator architectures, causing various types of electrically controllable shape changes. To achieve large actuation forces, a particular requirement for solid polymer films within such actuators is to combine exceptionally high dielectric strength with high mechanical yield strength (*26*). A few specific types of films, including commercial polymers within the classes of polypropylene and polyester, meet these requirements and are commonly used materials for fabricating nonbiodegradable actuators (*26*, *29*, *32*, *33*). There exist numerous biodegradable polymers that are designed for durable packaging and therefore feature sufficient mechanical yield strength for use in actuators: These include polylactic acid (PLA), polybutylene adipate terephthalate, and thermoplastic starches (*36*). However, these biodegradable polymers are not typically designed for electrical insulation, and little work has been done to investigate their electrical properties or suitability for electrostatic actuators that harness liquid dielectrics.

**Fig. 1.**
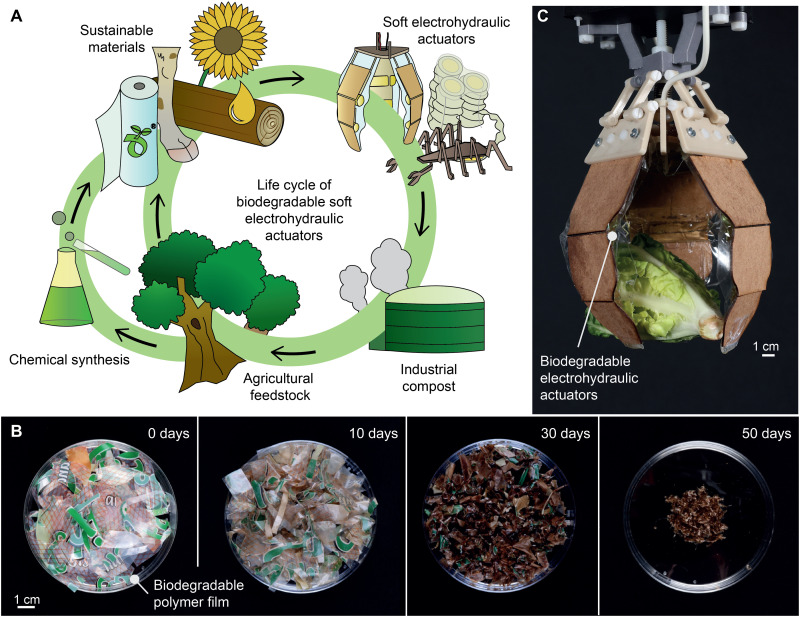
Biodegradable materials for sustainable electrohydraulic soft actuators. (**A**) A schematic life cycle of a biodegradable soft electrohydraulic actuator. (**B**) Biodegradable polyester blend (biopolyester) film disintegrating over the course of 50 days under industrial composting conditions. (**C**) A biaxially oriented polylactic acid (BOPLA)–based biodegradable gripper with fiber board segments grasping a lettuce head.

Here, we lay out a strategy to systematically determine mutually compatible materials systems to create fully biodegradable, high-performance soft electrostatic actuators that harness liquid dielectrics. Specifically, we showcase materials systems in the form of fully biodegradable hydraulically amplified self-healing electrostatic (HASEL) actuators, based on biodegradable polymer films that simultaneously display favorable mechanical strength and dielectric properties, ester-based liquid dielectric, and NaCl-infused gelatin hydrogel. Biodegradable HASELs are designed such that at the end of product life, their entirety can be composted into biomass at an industrial facility. The resulting biomass delivers nutrients for future plant growth, parts of which can eventually serve as raw material or be chemically synthesized into new materials for actuator production (Fig. 1A). For fully biodegradable HASELs, we used solid polymer films in the form of industrially compostable biaxially oriented PLA (BOPLA) or a biodegradable polyester blend (biopolyester; Fig. 1B). We show that the actuation performance of biodegradable HASELs is competitive with their nonbiodegradable counterparts, featuring the ability to operate at high electric fields of up to 200 V/μm and to reliably actuate for more than 100,000 cycles at maximum operational voltages. In addition, we identify pathways for improving specific materials properties for further advancing actuation performance of biodegradable HASELs. We lastly demonstrate an application of biodegradable HASELs in a gripper device that is readily compatible with commercial robot arms (Fig. 1C). Our results open up multiple avenues for future research not only for biodegradable soft actuator devices but also for projects that aim to make other types of electric field–driven technologies biodegradable.

## RESULTS

**Fig. 2.**
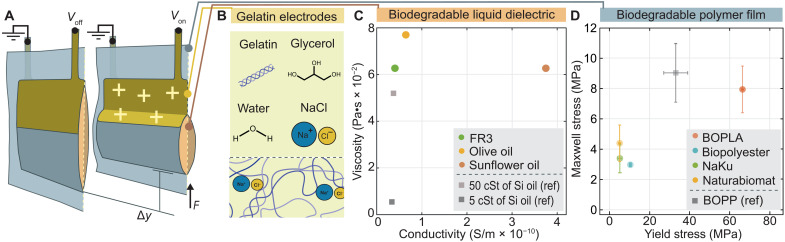
Evaluation of materials properties for biodegradable Peano-HASEL actuators. (**A**) Working principle of a Peano-HASEL (hydraulically amplified self-healing electrostatic) actuator when (left) voltage is turned off and (right) voltage is turned on. (**B**) Components of NaCl-infused gelatin hydrogel electrodes. (**C**) Dynamic viscosity plotted over conductivity for three biodegradable oils: ester-based FR3 liquid dielectric, olive oil, and sunflower oil [50 and 5 centistokes (cSt) of silicone oils as reference]. (**D**) Maxwell stress plotted over yield strength for four biodegradable thin films: BOPLA, biopolyester, and two starch blends from NaKu and Naturabiomat (see also table S1 for experimental values).

We chose to produce a particular geometry of HASELs called “Peano-HASEL” actuators as a biodegradable actuator model system due to manufacturing simplicity and reproducibility (Fig. 2A) (*29*). Peano-HASELs are rectangular plastic pouches filled with liquid dielectric and partially covered with flexible electrodes. Application of voltage across the electrodes causes liquid to redistribute, such that a linear contraction of the pouch is achieved. Before fabricating biodegradable Peano-HASELs, we first analyzed the mechanical and electrical properties of several polymer film and liquid dielectric candidates for actuator components. Gelatin hydrogel served as the sole biodegradable electrode candidate (Fig. 2B), which was based on previous work (*22*). For determining the influence of material combinations, we also studied the electrical properties of polymer films under the presence of electrodes and liquid dielectric. The highest performing biodegradable material systems were selected to fabricate Peano-HASELs, and their electromechanical performance was compared with nonbiodegradable counterparts.

### Biodegradable liquid dielectrics

The liquid dielectric contained in a HASEL influences its mechanical performance (*37*), largely through dynamic viscosity. A liquid dielectric with a high dynamic viscosity can prevent actuators from performing at high speeds due to the displaced medium being unable to flow and induce shape deformation at the rate of changing voltage conditions. Along with favorable dielectric properties, we therefore sought a liquid with low values for dynamic viscosity.

Typical liquid dielectrics used in HASELs include nonbiodegradable silicone oil and a readily biodegradable ester-based transformer oil called FR3 (*38*). Common vegetable oils also exhibit sufficient dielectric properties that qualify them as candidates for biodegradable HASELs (*24*).

We compared the conductivities and dynamic viscosities of three biodegradable liquid dielectrics—FR3, sunflower oil, and olive oil (Fig. 2C and fig. S1). We benchmarked biodegradable oil performance compared to 5 and 50 centistokes (cSt) of silicone oils that we used as a reference, which had dynamic viscosities of 5.4 × 10^−3^ and 5.2 × 10^−2^ Pa·s and conductivities of 3.38 × 10^−11^ and 3.72 × 10^−11^ S/m, respectively. Sunflower oil exhibited the lowest viscosity after silicone oil at 6.2 × 10^−2^ Pa·s but displayed a high conductivity of 37.1 × 10^−11^ S/m. Olive oil showed a lower conductivity of 6.45 × 10^−11^ S/m but a higher viscosity of 7.7 × 10^−2^ Pa·s. FR3 ultimately exhibited the most desirable combination of a low viscosity of 6.3 × 10^−2^ Pa·s and a low conductivity of 4.04 × 10^−11^ S/m, establishing it as the liquid dielectric of choice for the remainder of this work. Note, however, that olive and sunflower oils are interesting candidates for edible soft actuator applications.

### Properties of biodegradable polymer films

Biodegradable plastics have been studied for decades and designed with mechanical strength suitable for implementation into products like food packaging (*39*). For biodegradable HASELs, they would serve as the insulating shell that encapsulates liquid dielectric and provides structural support while bearing loads.

Conveniently, there exists a European Union certificate, “EN 13432/14995,” confirming the industrial biodegradability of certain commercial products that, once disposed of, achieve 90% absolute biodegradation within the course of 6 months (*40*). We used this certification to select four film candidates for fabricating into HASELs, which included 15-μm-thick BOPLA, 27-μm-thick biopolyester film, 12-μm-thick Naturabiomat starch blend, and a 28-μm-thick NaKu starch blend. Notably, biopolyester film was available in the form of preprinted grocery bags designed for carrot packaging and is therefore marked with colorful graphic designs. The breakdown strength of biopolyester did not vary markedly between regions containing or lacking prints, with a difference of only 4 V/μm for average values (table S1).

Figure 1B demonstrates biopolyester samples biodegrading in a climatized chamber while immersed in water-saturated compost soil, simulating industrial composting facilities (see fig. S2 for the setup). Samples after 30 days already showed advanced decomposition, and only traces were visible after 50 days. Apart from increased brittleness, reference biopolyester samples exposed to aerated deionized (DI) water under the same temperature conditions showed no signs of degradation after 50 days, indicating that microorganisms from the soil are necessary for initiating film biodegradation (fig. S3).

Mechanical tensile tests and standardized electrical breakdown tests [under American Society for Testing and Materials (ASTM) protocol D-149] and dielectric spectroscopy were conducted on biodegradable films to assess their yield strengths, dielectric strengths, and dielectric constants. We compared the performance of biodegradable films to that of a nonbiodegradable 20-μm-thick biaxially oriented polypropylene film (BOPP).

Tensile tests revealed that BOPLA has the highest yield strength among the four biodegradable films and even surpassed reference BOPP film, with an average strength of 66.4 ± 0.7 MPa. Biopolyester showed the next highest yield strength among biodegradable films with 10.3 ± 0.5 MPa, while the remaining two films both displayed approximately 5 MPa. All films including BOPP also exhibited anisotropic mechanical behavior (fig. S4), which was considered when fabricating actuators by ensuring that films were oriented to withstand maximal loading.

Standardized tests of dielectric strength were performed by ramping a 500 V/s voltage signal across samples until dielectric breakdown occurred (fig. S5). Reference BOPP reached the highest average dielectric strength at 680 V/μm (a voltage of 13.6 kV). BOPLA tolerated the highest electrical field before failure among all investigated biodegradable films at 559 V/μm (8.4 kV), which is likely attributed to its biaxial orientation, a process shown to typically increase dielectric strength of polymer films (*41*). Biopolyester film showed the next highest average dielectric strength of 213 V/μm (5.7 kV). The two starch blends from Naturabiomat and NaKu had similar average dielectric strengths of 199 and 191 V/μm, respectively (table S1).

Dielectric constant measurements were performed using an impedance spectroscopy setup, under 1 V root mean square AC voltage and a frequency range sweeping from a maximum of 1 MHz to a minimum of 1 mHz (fig. S6). BOPLA exhibited the lowest dielectric constant value among the biodegradable films of 2.87. Biopolyester displayed a constant of 5.43, and Naturabiomat and NaKu films displayed the highest constants of 10.46 and 12.57 at 1 Hz (see table S1 for 1-kHz values), respectively. BOPP films exhibited a lower dielectric constant than all biodegradable films, with a value of 2.19.

Using the above results for dielectric strength and dielectric constant, we next calculated the maximum Maxwell stress (proportional to the product of the dielectric constant and the square of the electric field) exerted across the films and plotted the results over their respective yield strengths (Fig. 2D). BOPLA exhibited the highest overall combination of yield strength (66.4 ± 0.7 MPa) and maximum Maxwell stress (7.9 ± 1.5 MPa) of all biodegradable film candidates, deeming it the most promising actuator material. Among the other three films, we also decided to use biopolyester film as a biodegradable comparison material to BOPLA due to it having the next highest yield strength (10.3 ± 0.1 MPa) as well as a highly consistent dielectric performance compared to Naturabiomat and NaKu films, which had larger SDs of 0.9 and 1.2 MPa, respectively (table S1).

**Fig. 3.**
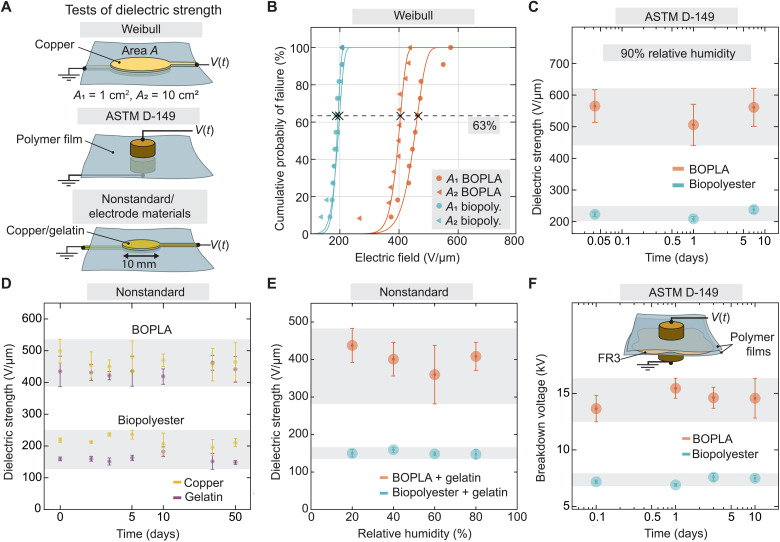
Evaluation of dielectric strength of BOPLA and biopolyester films. (**A**) Experimental setups to test dielectric strength included: (top) circular evaporated copper electrodes used for Weibull tests, (middle) standard ASTM D-149 cylindrical brass electrodes, and (bottom) nonstandard 10-mm-diameter circular electrodes made from NaCl-infused gelatin hydrogel or evaporated copper as reference material. (**B**) Weibull distribution for BOPLA and biopolyester films with circular copper electrodes with areas *A*_1_ = 1 cm^2^ and *A*_2_ = 10 cm^2^. (**C**) Dielectric strength of BOPLA and biopolyester exposed to 90% relative humidity over the course of 10 days. (**D**) Dielectric strength of BOPLA and biopolyester using copper and hydrogel electrodes and stored under ambient conditions was tested between 0 and 50 days after fabrication. Consult table S2 for additional details. (**E**) Dielectric strength of BOPLA and biopolyester films with gelatin hydrogel electrodes, exposed to 20, 40, 60, or 80% relative humidity for 1 day. (**F**) Standardized breakdown tests performed across two films separated by a thin layer (0.05 ml) of FR3 under ambient conditions.

Inhomogeneities within materials often are the cause of premature dielectric breakdowns due to the creation of intensified electric fields within localized material regions (*42*). Inhomogeneities within BOPLA and biopolyester films were initially investigated by performing tests of dielectric strength using 300-nm-thick evaporated copper electrodes of two different circular areas of 1 and 10 cm^2^ and then plotting their Weibull cumulative probability distribution of failure (Materials and Methods, Fig. 3A, and table S2). Characteristic dielectric strength was taken as the electric field at which 63% of the samples experienced dielectric breakdown (*43*). BOPLA samples exhibited a noticeable change in dielectric strength with a change in electrode area, with characteristic dielectric strength shifting from 465 V/μm for 1-cm^2^ electrodes to 405 V/μm for 10-cm^2^ electrodes. Biopolyester samples did not show a strong area-dependent breakdown behavior, with a slight shift from 197.9 to 187.3 V/μm (Fig. 3B).

To confirm the presence of inhomogeneities, we investigated the films with optical microscopy, which revealed a significant number of visible inhomogeneities in BOPLA—with maximum recorded diameters of 11 μm using optical microscopy. Biopolyester did not show such inconsistencies within its volume, although it exhibited scratches likely produced during film production and processing (fig. S7). Further investigations of film surface morphologies using scanning electron microscopy, atomic force microscopy, and profilometry confirmed a high density of surface inhomogeneities (fig. S8). Across a sample area of 0.25 mm^2^, both films presented a total surface height variation of approximately 700 nm. Notably, although BOPLA showed an overall smoother surface texture than biopolyester, it additionally exhibited more prominent surface inhomogeneities, with cavities with depths of up to 0.3 μm and protrusions of up to 1.4 μm from the film surface. The above microscopy and profilometry results, together with findings from the Weibull plot in Fig. 3B, further corroborate that physical inhomogeneities in biodegradable films play a large role in electrical insulation performance.

Because humidity is one of the parameters known to accelerate material biodegradation (*44*), we hypothesized that moist environments would shorten the useful lifetime of biodegradable films as a material for soft electrohydraulic actuators. Using ASTM D-149 standards, we therefore measured the dielectric strength of films immediately after being stored for different durations under 90% relative humidity at 23°C. Films kept in a climate chamber over the course of 10 days showed negligible change in breakdown performance, suggesting that environmental humidity does not substantially influence dielectric breakdown properties of either biodegradable film (Fig. 3C).

### Influence of electrodes on biodegradable polymer films

We have developed a nontoxic, biodegradable NaCl-infused gelatin hydrogel that can be directly blade-casted onto actuators (fig. S9), using a recipe that we adjusted from previous work (*22*). These gelatin hydrogel electrodes, which mainly consist of water, glycerol, and food-grade gelatin derived from animal collagen (Fig. 2B), are readily dissolved under warm water and tunable in thickness and stiffness. Gelatin hydrogels equilibrate in moisture with different humidity environments (fig. S10), display high ionic conductivity that remains consistent over time (fig. S11), and exhibit suitable adhesion to biodegradable films (fig. S12) while staying well adhered under repetitive bending stresses (movie S1).

A standardized test of dielectric strength would be impractical for determining the influence of gelatin hydrogel electrodes on biodegradable films, since ASTM D-149 conditions require specific electrode materials, geometries, and weights that cannot be realized when using hydrogel electrodes. We therefore defined custom “nonstandard” tests of dielectric strength (Fig. 3A).

For our nonstandard electrodes, we cast 225-μm-thick gelatin hydrogels on either side of single sheets of film with a 10-mm-diameter circular geometry, across which we applied a ramped 500 V/s voltage signal until dielectric breakdown occurred. We repeated the measurements using 300-nm-thick evaporated copper electrodes with the same circular geometry.

We observed a decrease in dielectric strength of films in nonstandard breakdown tests for both hydrogel and copper electrode conditions, compared to when using the standardized ASTM D-149 setup. The average dielectric strength of BOPLA lowered from 599 to 499 V/μm when comparing ASTM test results to those from copper electrodes, while for biopolyester, this value dropped from 248 to 218 V/μm, respectively (Fig. 3D). In the case of BOPLA, a reduction in dielectric strength can be partially attributed to the larger surface area of the 10-mm-diameter electrodes (compared to the 8-mm-diameter electrodes with curved edges used for ASTM tests), which previous Weibull results predicted would lower breakdown strength.

BOPLA exhibited an average breakdown strength of 435 V/μm when using gelatin hydrogel electrodes, an additional drop of 13% compared to samples exposed to copper electrodes, while biopolyester exposed to hydrogel yielded 159 V/μm (Fig. 3D), a drop of 27%. We speculate that ionic species from the hydrogel may be driven into the dielectric layer by high electric fields; conduction types of the electrodes (electronic versus ionic) could influence diffusion mechanisms across the dielectric layers, consequently changing their insulating properties (*45*).

We measured the dielectric strength of biodegradable films that were exposed to gelatin hydrogel and copper electrodes over the course of 50 days under ambient conditions of 23°C and 40% relative humidity (Fig. 3D). Notably, there were negligible changes in dielectric strength over 50 days for all electrode film combinations, again supporting the hypothesis that NaCl-infused gelatin hydrogel electrodes are not influencing the dielectric properties of films under ambient conditions, but only when exposed to high electric fields.

In addition to studying film electrode compatibility under ambient conditions, we measured the dielectric strength of films attached to gelatin hydrogel under different humidity conditions. Glycerol is a key hygroscopic ingredient in gelatin hydrogels that is particularly sensitive to humidity, which helps to prevent hydrogels from drying under lower humidities (Fig. 3E) (*46*). Hydrogel-covered film samples were stored under 23°C and either 20, 40, 60, or 80% relative humidity for 24 hours, before testing their dielectric strength. The results for both films did not indicate a statistically significant correlation between relative humidity and dielectric strength of hydrogel-covered films. The large errors displayed by BOPLA samples in comparison to biopolyester are likely linked to their high density of physical inhomogeneities, which would align with results shown from previous Weibull tests.

### Influence of liquid dielectric on biodegradable polymer films

Last, we examined whether FR3 oil influences the electrical performance of biodegradable films. We stacked two layers of biodegradable films and inserted between them a 0.05-ml drop of FR3. We used a standardized breakdown setup but analyzed breakdown voltage, instead of electric field, due to difficulty in assessing the oil thickness separating the two film layers. Similar to the ratio of breakdown fields between BOPLA and biopolyester in previous standardized tests, BOPLA broke near twice the breakdown voltage of biopolyester, with an average of 13.7 ± 1.2 and 7.18 ± 0.14 kV for the two respective films. We repeated tests with the stacked sample stored for 10 days under ambient conditions and found negligible differences in breakdown voltages, suggesting that films can remain in contact with FR3 for prolonged periods without risk of degradation (Fig. 3F).

### Biodegradable Peano-HASEL actuators

**Fig. 4.**
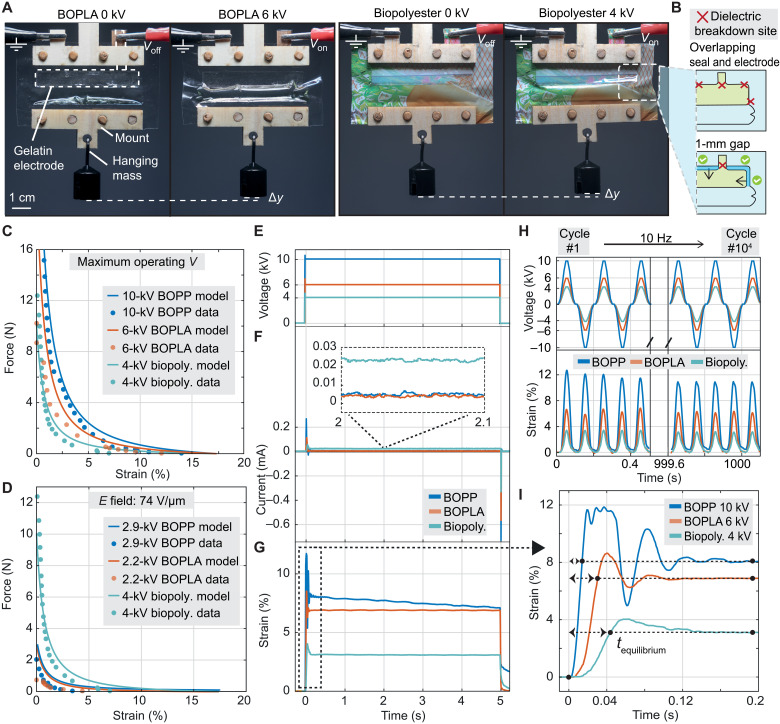
Mechanical characterization of Peano-HASEL actuators made from biopolyester, BOPLA, and BOPP films with NaCl-infused gelatin hydrogel electrodes. (**A**) Single-pouch Peano-HASEL actuators with 60 mm wide–by–20 mm tall geometries made from (left) BOPLA and (right) biopolyester films. (**B**) NaCl-infused gelatin hydrogels were cast with a 1-mm gap from heat seals, which effectively minimized premature dielectric breakdown. (**C**) Force-strain plots of actuators at maximum reliable operating voltage and (**D**) under the same electric field of 74 V/μm. (**E**) Constant DC signals at maximum reliable operating voltages that were applied across actuators made from each material, along with (**F**) respective output current and (**G**) actuator strain over time while bearing a 50-g mass. (**H**) (Top) Snapshots of reversing polarity 10-Hz sinusoidal waves with an amplitude at maximum reliable operating voltages at cycle 0 and cycle 10000, along with (bottom) correlating actuator strain while bearing a 50-g mass. (**I**) Dynamic strain of actuators bearing a 50-g mass in response to a square wave signal at maximum reliable operating voltages. *t*_equilibrium_ is the time required for actuators to reach an equilibrium actuation strain.

Biodegradable Peano-HASEL actuators were manufactured from BOPLA and biopolyester, gelatin hydrogel electrodes, and FR3 oil. To compare biodegradable to reference nonbiodegradable actuators, we exchanged the insulating shell for BOPP as a reference material while keeping other materials and manufacturing steps consistent (fig. S13). We compared the actuation performance of linearly contracting, 60 mm wide–by–20 mm long single-pouch Peano-HASEL actuators (Fig. 4A and fig. S14). We left a 1-mm gap between hydrogel electrodes and heat seals (Fig. 4B), a design that diminished premature dielectric breakdown effects (table S2, last column).

We investigated the mechanical performance of biodegradable actuators in relation to their BOPP counterparts by measuring the following performance parameters: force-strain relationship, peak strain rate, power consumption, and lifetime. The force-strain relationship of an actuator reveals its overall usefulness across a range of loading conditions and has been successfully modeled for Peano-HASELs in previous work, which takes into consideration oil fill, actuator geometry, dielectric constant of polymer film, applied voltage, and mechanical loading condition (*47*). We included this established model into our force-strain plots, along with an experimentally determined multiplicative factor of 0.7 for each model to account for the electrode seal gaps. These gaps come with a decrease in electrode width and length compared to standard geometries, causing force to decrease, since the force output of Peano-HASELs is proportional to electrode width.

Biodegradable Peano-HASELs were subjected to an experimentally validated maximum reliable operating voltage, which we set as approximately 60% of the observed actuator breakdown voltage: Reliable operating voltages were 6 kV (~200 V/μm) for BOPLA and 4 kV (74 V/μm) for biopolyester. In the case of BOPP, electrical arcing through air was the limiting factor; hence, we limited the voltage to 10 kV (~250 V/μm). We applied square wave voltages at 0.5 Hz while under loading conditions, spanning 0 to 20 N (Fig. 4C).

Peano-HASELs made from BOPLA under 6 kV exhibited an average free strain of 12%, an average blocking force just below 9 N, and a specific energy of 1.72 J/kg. Biopolyester Peano-HASELs under 4 kV exhibited a free strain of 7% and an average blocking force of 12 N with a specific energy of 0.96 J/kg. The reference BOPP Peano-HASELs under 10 kV exhibited 14.4% strain and specific energy of 4.43 J/kg. BOPP Peano-HASELs showed the highest force and strain due to experiencing a higher Maxwell stress. Both biopolyester and BOPP Peano-HASELs closely matched models. BOPLA had a noticeably lower performance than predicted, which we speculate is because of the higher mechanical stiffness of BOPLA that could constrain actuation.

We analyzed a second scenario, where we applied different voltages for each actuator such that they experienced the same electric field of 74 V/μm (Fig. 4D), which correlated to 4 kV for biopolyester, 2.9 kV for BOPP, and 2.2 kV for BOPLA. In this case, biopolyester Peano-HASELs showed superior performance over other actuators, with 7% free strain and 12-N blocking force, much of it due to having a larger dielectric constant of 5.43 (BOPP = 2.2 and BOPLA = 2.87 at a frequency of 1 Hz). BOPP Peano-HASELs in comparison experienced free strain of 3.5% and a blocking force of less than 2 N, while BOPLA actuators exhibited a free strain of 4.5% with a blocking force of approximately 0.8 N.

The working principle of a HASEL allows for a catch state (*26*), but in reality, they require some power to compensate for charge leakage and corona discharge effects. As a performance metric, we measured the current leakage across biodegradable and BOPP-based Peano-HASELs as they were exposed to constant DC voltage under maximum reliable operating voltages (Fig. 4E). As shown in Fig. 4F, over the course of 5 s, BOPLA actuators, similar to BOPP actuators, displayed minimal current leakage (<5 × 10^−3^ mA), while biopolyester actuators exhibited a visibly larger average charge leakage of 2.3 × 10^−2^ mA (see fig. S15 for power consumption of each actuator). However, when plotting resulting strains (Fig. 4G), both biopolyester and BOPLA-based Peano-HASELs maintained a more consistent strain compared to BOPP actuators, which displayed decrease of strain over time due to what is believed to be charge retention effects (*29*); over the course of 5 s, the BOPP Peano-HASEL decreased by 14% from its initial equilibrium strain, while both BOPLA and biopolyester-based Peano-HASELs deviated by less than 0.1% from their initial equilibrium strain, which indicates superior controllability compared to BOPP actuators.

The lifetime of actuators indicates whether they are useful for extended periods of time. This metric could be a concern for biodegradable actuators, since biodegradability inherently suggests the ability of the actuator to deteriorate over time. We therefore tested the lifetime of biodegradable Peano-HASELs by applying across them a reversing polarity sinusoidal voltage signal at both 5 and 10 Hz with maximum operating voltage as the signal amplitude, until breakdown occurred. For each frequency and material, measurements were repeated with two individual actuators, and the maximum lifetime was recorded. At 10 Hz, BOPLA Peano-HASELs survived 22,000 cycles, while at 5 Hz, BOPLA Peano-HASELs exhibited a premature breakdown after 1600 cycles. These relatively low lifetime numbers could be stemming from the number of inhomogeneities inside BOPLA films (see discussion above), which could be improved with materials advancement. On the other hand, at both 10 and 5 Hz, both biopolyester Peano-HASELs survived past 100,000 cycles, at which point we discontinued testing. Figure 4H shows initial strain at 10 Hz and 1000 s later (correlating to 10,000 cycles), indicating that the strain for BOPLA and biopolyester-based actuators remains within approximately 95 and 93% of its strain in the first initial actuation cycles, respectively.

Dynamic effects are an important consideration for soft actuators, especially in high-speed applications. We measured the peak strain rates and rise times of biodegradable Peano-HASELs in comparison to BOPP Peano-HASELs at maximum reliable operating voltages (Fig. 4I). Peak strain for BOPLA Peano-HASELs was 494%/s, approaching the maximum peak strain for mammalian skeletal muscle (500%/s) (*48*). Biopolyester actuators neared their equilibrium position at a peak strain rate of 175%/s; this value is expected to increase if the dielectric strength of biopolyester were to increase, since a higher applied electric field would induce a faster actuation response (assuming operation under an inertia-driven regime) (*37*). In comparison, BOPP Peano-HASELs exhibited a peak strain rate of 981%/s.

### Demonstration of application: Biodegradable SES gripper

**Fig. 5.**
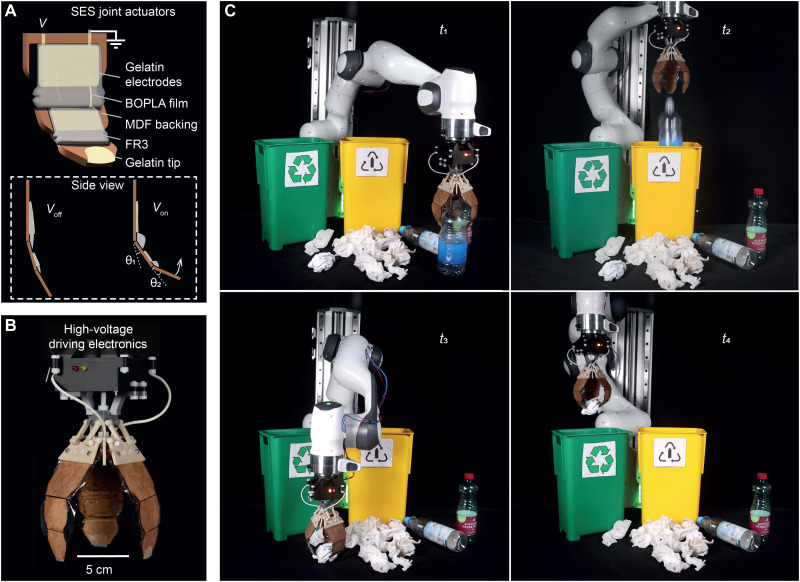
SES joint-based biodegradable gripper. (**A**) One gripper unit consists of two SES (spider-inspired electrohydraulic soft-actuated) joints mounted on a medium-density fibreboard (MDF) backing with a gelatin end effector on its tip. (**B**) Three gripper units are positioned equidistant from each other to form a gripper. Biodegradable PLA is used to three-dimensionally (3D) print the gripper mount. (**C**) Demonstration of a gripper mounted onto a commercial robot arm, disposing of plastic bottles and crumpled paper balls into designated waste containers over the course of time stamps t_1_-t_4_.

Sustainable robotics are particularly useful for scenarios demanding single-use deployment, where simply disposing of a traditional robotic component would otherwise come with a high expense both to users and the environment. This includes garbage-collecting robots, which may be exposed to toxic substances that could spread contamination with additional usage if deployed more than once. To demonstrate the potential of biodegradable soft electrohydraulic actuators for use in such robotic applications, we incorporated them into a versatile soft-actuated robotic gripper. We fabricated a fully biodegradable gripper consisting of three spider-inspired electrohydraulic soft-actuated (SES) joints that picked up and released objects of different dimensions (Fig. 5A) (*28*). Individual SES joints consisted of soft electrohydraulic actuators made from gelatin hydrogel, BOPLA, and FR3, which were adhered to recycled medium-density fiber board backings. A compliant end effector was made from gelatin hydrogel, which was coated with talc powder to prevent sticking (Fig. 5B and fig. S16). The gripper was attached to a three-dimensionally (3D) printed mount made from PLA—an industrially compostable polymer—and attached to a programmable commercial robotic arm. In a programmed sequence, the robotic arm approached objects [polyethylene terephthalate (PET) bottle and a crumpled paper], grasped objects upon application of voltage, and held them under constant DC voltage until the robotic arm placed them above the respective garbage can, at which point turning off voltage released the objects (Fig. 5C and movie S3). We additionally characterized the normal tip force of a single pincer unit comprising an SES gripper, which can be exploited to grasp an object, and measured a maximum normal force of 0.21 N at 6 kV (figs. S17 and S18 and movie S4).

## DISCUSSION

Exploring plastics intended for commercial packaging, we have shown that some biodegradable materials systems are suitable for use in HASEL actuators. HASELs made from gelatin hydrogels, ester-based liquid dielectrics, and biodegradable polymer films can reliably operate under high electric fields of 200 (for BOPLA) and 74 V/μm (for biopolyester). Standardized breakdown tests of films indicate that biodegradable polymers are able to withstand even higher electric fields, highlighting the opportunity that future research can further optimize the materials system to reach even higher actuator performance. However, when operated at lower electric fields, biodegradable actuators already show attractive force-strain performance, exhibiting nearly 83% of the free strain of Peano-HASELs made from BOPP. As verified with biopolyester, biodegradable films can exhibit higher dielectric constants than some commonly used nonbiodegradable plastics, allowing for actuators to achieve the same electromechanical performance using lower electric fields. The two starch-blended films that were not tested for actuator performance, Naturabiomat and NaKu films, have particularly high dielectric constants of 10.46 and 12.57; with further optimization of their yield strength and dielectric robustness, there is potential for fabricating biodegradable HASEL actuators that operate under even lower voltages than those from biopolyester.

Efforts to develop biodegradable films specifically for dielectric applications could resolve challenges that we mentioned previously: biopolyester film lacked a sacrificial heat seal layer, resulting in film weakening in the sealed region. While implementing a 1-mm gap between electrode edge and heat seals of actuators minimized the probability of breakdown, failure would still occur at one of two sites where electrode leads and heat seals overlapped. Solutions for preventing premature breakdown could include developing biopolyester films with a sacrificial heat-sealing layer, which would allow use of higher driving voltages. Materials improvements for BOPLA should focus on reducing regions of inhomogeneity; this could involve using techniques for manufacturing capacitor-grade films, such as the use of high-purity polymer pellets and advanced filtration before biaxial orientation of films (*49*). In addition, it is important to take substantial care when handling films before manufacturing into an actuator to avoid adding physical defects.

Although in our case we introduced a materials system specifically for fully biodegradable HASEL actuators, the same materials system could potentially be used in other types of soft electrostatic actuators harnessing liquid dielectrics, such as electroribbons (*30*) and hydraulically-amplified taxels (HAXELs) (*33*). The use of biodegradable materials systems for such actuators, in this way, opens up previously unknown avenues to design sustainable robotic systems.

## MATERIALS AND METHODS

### Materials

Polymer films were used as received from commercial packaging industries and included the following: 27-μm-thick printed NATURAPACKAGING biopolyester (received from Naturabiomat GmbH, Austria), 12-μm-thick Naturabiomat starch-blend plastic (received from Naturabiomat GmbH, Austria), 28-μm-thick NaKu starch-blend (received from NaKu e.U., Austria), 15-μm-thick BOPLA (Nativia NTSS, distributed by Pütz GmbH + Co. Folien KG, Germany), and 20-μm-thick “HSF5114H” BOPP (Multiplastics Europe Ltd., UK). Liquid dielectrics were also used as received and included Envirotemp FR3 ester-based transformer liquid (Cargill Inc., USA), cold-pressed sunflower oil and olive oil (BioBio Co., Germany), and 5 and 50 cSt of silicone oils (Sigma-Aldrich Co., Germany). Gelatin hydrogel electrodes consisted of gelatin powder with a bloom factor of 260 (Ewald-Gelatine GmbH, Germany), NaCl [American Chemical Society (ACS) reagent 99+%, Acros Organics B.V.B.A., Belgium], DI water, and glycerol (Rotipuran ≥99.5%, Carl Roth GmbH + Co. KG, Germany).

### Characterization of liquid dielectrics

The rheological properties of the liquid dielectrics were determined using a modular advanced rheometer [HAAKE Modular Advanced Rheometer System (MARS) III, Thermo Fisher Scientific, USA] equipped with a coaxial cylinder geometry with an inner diameter of 32 mm [CC25 Deutsches Institut für Normung (DIN) Ti and CCB25 DIN, Thermo Fisher Scientific, USA]. The instrument was controlled using the software HAAKE RheoWin (Thermo Fisher Scientific, USA). Samples were placed in the cup at room temperature 21°C with a gap of 5.3 mm between the cylinder and cup, and the dynamic viscosity was measured as a function of shear rate sweeping from a low frequency of 0.1 s^−1^ to a high frequency of 1000 s^−1^.

The dielectric properties of the dielectric fluids were determined using a high-resolution dielectric spectrometer (Novocontrol Alpha-A frequency analyzer, Germany) equipped with a liquid sample cell (BDS 1308), 100-μm fused silica electrode spacer, and measured along the range of 1 mHz to 70 kHz at a fixed temperature of 21°C.

### Simulating the industrial biodegradation of biopolyester

For simulating the conditions of an industrial biodegradation facility, three 300-ml plastic bottles were filled with 170 g of water-saturated compost soil, into which we inserted 1 ± 0.015 g of dumbbell-shaped biopolyester samples [dimensions from standardized tensile tests: ISO527-2:1996(5A)]. One additional bottle, containing 1 g of samples and 200 ml of DI water, was used as reference. The bottles were connected in series using caps with holes for tubing (fig. S2). The tubing was placed such that water-saturated air flowed through all bottles with samples to maintain soil hydration. The bottles were kept in a box inside of an oven at 58°C. A bottle was removed from the setup after 10, 30, and 50 days, respectively. The biopolyester samples were separated from the soil, washed, and dried.

### Mechanical characterization of polymer films

Uniaxial tensile tests were performed using a uniaxial tensiometer (Zwick Roell Z005, 100-N load cell, Germany) on standardized dumbbell-shaped geometries [ISO 527-2:1996(5A)] laser-cut from polymer films (Speedy 300 Flexx, Trotec, Austria). The tests were performed at a strain rate of 30 mm min^−1^ and a preload of 0.1 N. Tests were performed on three specimens of each sample material. Yield strength of films were determined by fitting a linear slope with an *x* offset of 0.2% strain to the area of linear regime within each film’s tensile test results. The *y* value at the region where the fit and tensile test results crossed was determined as the yield strength of the film. The traditional approach of using a local stress maximum before plastic deformation was not possible, as some samples lacked a local maximum.

### Electrical characterization of polymer films

For impedance spectroscopy measurements, 300-nm-thick circular copper electrodes with a diameter of 10 mm were evaporated on both sides of polymer films. The samples were then put in a shielded aluminum enclosure and connected to an impedance analyzer (Novocontrol Alpha-A Analyzer, Germany). Measurements were performed at 1 *V*_rms_ ac voltage and frequencies of 10^−3^ to 10^6^ Hz.

Breakdown voltage of different biodegradable films was determined according to the standard ASTM D-149. Film samples with dimensions of 30 by 30 mm were cut out and sandwiched between two circular brass electrodes with diameters of 8 mm, round edges, and an upper electrode weighing 50 ± 2 g. Voltage was ramped from 0 V upwards at a rate of 500 V/s until breakdown was detected using a custom LabVIEW program. Tests were performed on seven specimens of each sample material.

For determining the Weibull statistics of defect-driven breakdown displayed by polymer films, we fitted a cumulative distribution function to our Weibull experiments in Fig. 3B using the MATLAB “ezfit” toolbox and fitting with the following equation

$$1-e^{-{\left(x/\gamma \right)}^{a}}$$

where *x* is the varying electric field, *a* represents the shape parameter, and γ is the characteristic breakdown strength of the material, taken as the breakdown field where 63% of our samples experienced electrical failure (*43*). Table S2 lists the equations with values that best represent the Weibull breakdown distribution for BOPLA and biopolyester films.

Standardized breakdown tests were conducted to investigate the influence of humidity on the breakdown voltage of the dielectric films, which were previously stored in a climate chamber (23°C, 90% relative humidity) for 0, 1, 2, 3, or 10 days before testing. Tests were performed on at least five specimens of each sample material.

For investigating the influence of gelatin and reference copper electrodes on films over time, 10-mm diameter electrodes were applied to polymer film samples, which were then stored for 0, 1, 2, 10, 30, or 50 days under ambient conditions (23°C, 40% relative humidity) before undergoing breakdown tests. Tests were repeated at least five times.

The influence of gelatin electrodes on films under different ambient conditions was investigated by exposing biopolyester and BOPLA films, with 10-mm diameter gelatin electrodes applied on both sides, to 23°C and relative humidities of 20, 40, 60, or 80% for 24 hours. Breakdown tests of samples followed. Tests were repeated at least seven times.

### Optical characterization of the polymer films

Surface structure and morphology were visualized via optical microscopy, scanning electron microscopy, profilometry, and atomic force microscopy. Optical microscope (Eclipse LV100ND, Nikon, Japan) images were taken with magnifications of ×100 and ×400.

Scanning electron microscopy (JSM-6360LV, JEOL GmbH, Germany) was conducted with an acceleration voltage of 7 kV and at a vacuum pressure of 9 × 10^–5^ mbar.

Profilometry (DektakXT, Bruker Corporation, USA) was controlled with the software “Vision64” with a range of 6.5 μm, a radius of 12.5 μm, a stylus force of 2 mg, a length of 500 μm, and a map resolution 1 μm per trace and performed over 500 traces per sample with a trace duration of 5 s.

Atomic force microscopy (Innova, Bruker Corporation, USA) was performed with tapping/contact mode with settings of 4.699 V, Proportional: 0.5 V, Integral: 0.4 V, and Differential: 0.0 V and controlled with the software NanoDrive.

### Preparation and casting of gelatin electrodes

Gelatin electrodes were fabricated according to a procedure that was previously published (*22*). To establish ionic conductivity in the gelatin, 1 g of sodium chloride was quickly mixed into 4 ml of DI water. Next, 18 g of glycerol was added to increase the content of bound water in the hydrogel. The mixture was mixed at 400 revolutions per minute (rpm) using a planetary mixer (ARV-310PCE, THINKY, Japan). Next, 4 g of gelatin powder was added and again mixed at 400 rpm. After swelling overnight at room temperature, the mixture was heated in an oven at 50°C for 1 hour and stirred in a planetary mixer under vacuum of 35.9 kPa. The last step ensured a homogeneous gelatin precursor without any air bubbles, ready for casting onto films. The polymer films were adhered to a flat surface before gelatin casting. Hydrogel precursor was heated at 65°C until it formed a liquid and blade-casted on the heat-sealed film through a 225-μm-thick plastic mask to form gelatin electrodes. After applying gelatin, a 10-μm-thick bioplastic film strip was used to cover the gelatin for easier handling of the film. The film was then flipped to the opposite face, on which gelatin casting was repeated. The gelatin was given 1 day of curing on film before testing, which allowed for water content to adjust to ambient conditions. For testing reactivity of electrodes with films, we considered the day after curing as the 0th day condition.

### Characterization of gelatin electrodes

To investigate the typical weight change of gelatin hydrogel electrodes, samples were put on a precision scale (AEJ 200-4CM, Kern & Sohn GmbH, Germany) inside the climate chamber at 23°C and relative humidities of 20, 40, and 60%. The samples were produced in the same way as other electrodes with dimensions of 58 by 24 mm. They were not covered after fabrication and immediately transferred to the scale. Weight was recorded every second for 8 hours.

Peel tests were performed to investigate the adhesion properties of the gelatin hydrogel on BOPLA and biopolyester films. Gelatin hydrogel samples with dimensions of 120 by 30 by 2 mm were produced by casting them into custom molds. As shown in fig. S12A, the mold consisted of two base plates (base 1: 70 by 50 mm and base 2: 90 by 50 mm) made from 5-mm-thick polymethyl methacrylate (PMMA). A single sheet of biodegradable film was attached to base 2 using an adhesive (3M 467MP, 3M Austria GmbH, Austria), whereas base 1 was covered with a sheet of 12-μm-thick PET that was not attached to the plate. A 2-mm-thick PMMA plate, which had sample dimensions (120 by 30 mm) cut out of its middle, was placed on top of the base plates. The PMMA served as a template for casting hydrogel directly on top of biodegradable films (base 2) while creating a loose section on top of PET (base 1).

Gelatin hydrogel samples were prepared according to one of two conditions: (i) The hydrogels top surfaces were covered with PET sheets (which served to prevent gelatin elongation during the peel test) immediately after casting or (ii) were left exposed to their ambient environment for 24 hours before coverage, after which peel testing followed. Before the experiment, all mold parts except for base 2 were removed. This plate was mounted on a movable platform, while the loose section of hydrogel was clamped to a uniaxial tensiometer (Zwick Roell Z005, 100-N load cell, Germany) in a 90° angle with respect to the base plate (fig. S12B). Tests were repeated at least five times per film for each preparation condition. The samples were peeled off at a rate of 50 mm min^−1^. The resulting peeling force *F*_P_ between biodegradable films and gelatin hydrogels was used to calculate their debond energy. To do so, we subtracted the weight force *F*_W_ of the unbonded film hydrogel region from the peeling force and normalized the result by the gelatin sample width *w*. Debond energy is the average over an integral of the entire length *l*, starting at a point where the force has stabilized

$$F_{D}=\frac{1}{l}\int \frac{F_{P}-F_{w}}{w}dl$$

### Characterizing the conductivity of gelatin electrodes

Gelatin hydrogel electrodes promote ionic conductivity under voltage. We measured their resistivity using four-point probes. This was done by applying a voltage with a power supply (Rohde & Schwarz NGE100, Germany) across 10-mm-wide, 175-μm-thick gelatin hydrogel samples and measuring current on the ground side with an electrometer (Keithley 6514, USA) while measuring the voltage drop with a multimeter (Fluke 787B ProcessMeter, USA) across the gelatin hydrogel at a prescribed distance of 100 mm (see fig. S11 for setup and results). We applied a sufficiently high voltage of 30 V across samples, which compensated for the effects of smaller electrochemical potentials generated by ions accumulating near probes. Using the relationship

$$\rho =\frac{V}{I}*\frac{A}{l}\;[\text{Ω}\;\cdot m]$$

where *V* is the measured voltage, *I* is the measured current, *A* is the cross-sectional area of the sample, and *l* is the distance of sample across which the voltage drop is measured, we could then obtain the resistivity of the gelatin hydrogel. The resulting resistivity value could then be used to calculate molar conductivity, which we calculated to be 4.47 × 10^−2^ S cm^2^/mol (a conductivity of 191.4 × 10^−3^ S/cm) for fresh gelatin hydrogels containing 4.28 M of NaCl and 4.38 × 10^−2^ S cm^2^/mol (a conductivity of 187.3 × 10^−3^ S/cm) for the same batch of gelatin hydrogels exposed for 24 hours to ambient environmental conditions (40% relative humidity, 23°C). We repeated tests on three gelatin samples per condition.

### Fabrication of Peano-HASEL actuators

To determine parameters for heat sealing biopolyester and BOPLA films, we heat-sealed lines across two layers of film, each drawn with varying temperature, speed, and sealing tip force conditions. We used a Zwick tensile machine to measure the peeling strength of each line and eliminated any parameters that resulted in delaminated films. For the parameters that did not delaminate, next, we applied a 10-mm-diameter circular hydrogel electrode across a newly sealed line, across which voltage was ramped from 0 to 10 kV with a 500 V/s ramp rate until permanent failure was observed. Tests were repeated seven times, and we considered lines that withstood the highest voltage as the best heat-sealing parameter for a given polymer film.

Single-pouch Peano-HASEL actuators were manufactured using established methods (*31*) with heat seal dimensions of 61-mm width by 21-mm height and electrode dimensions of 58-mm width by 9-mm height. Heat seals were made using a 1-mm-diameter machined metal tip attached to a heating element and mounted to a preprogrammed Computerized Numerical Control (CNC) router (Carbide 3D Shapeoko XL, USA). Heat seal settings for BOPLA included a sealing tip temperature of 200°C, a sealing speed of 150 mm/min, and a sealing tip force of 1.33 N. Settings for biopolyester were 160°C, 500 mm/min, and 1.02 N, respectively. Settings for BOPP were 250°C, 300 mm/min, and a sealing tip force of 1.33 N, respectively.

We used electrodes geometries for all actuators with a 1-mm gap between seal line and electrodes (fig. S14), which effectively diminished premature dielectric breakdown across biopolyester and BOPLA actuators. Actuators were filled using a syringe with 1.9 ml of FR3, which was measured using a custom 3D printed syringe stopper that insured consistent fill between actuators.

Acrylic laser-cut mounts were used to hang actuators from a horizontal beam and to distribute loads evenly when applying a hanging mass below. Acrylic was used due to a high load tolerance for mechanical testing. For low-loading demonstrations, such as in Fig. 4A, we replaced the acrylic with thin wood, which demonstrates the possibility of manufacturing biodegradable mounts in addition to the actuating component.

### Electromechanical characterization of Peano-HASEL actuators

Voltage was applied across actuators using a Trek high-voltage amplifier (Trek 610), which amplified voltage signals inputted by a data acquisition (DAQ) device [USB-6212, National Instruments (NI), USA]. The DAQ was controlled using a custom MATLAB program.

Force-strain data (Fig. 4, C and D) of single-pouch Peano-HASELs were collected using a dual-mode lever system (Aurora Scientific 310C-LR, Canada). Zero- to 20-N force were applied in logarithmic steps to single-pouch Peano-HASELs under cyclic 0.5-Hz ramped-square reversing-polarity voltage conditions. We averaged the displacement of actuators for a given load across three voltage cycles. Strain measurements were read using NI DAQ and processed with a custom MATLAB program. We averaged the performance of two actuators for each material test (BOPLA, biopolyester, and BOPP) and used a Savitzky-Golay filter to remove displacement peaks due to inertial effects from strain analysis. For biopolyester actuators, we noticed an initial phase of actuators “wobbling” during actuation, which seems to be related to an inhomogeneous actuation phenomenon studied in previous work (*50*). This wobbling behavior dissipated with time (movie S2); we therefore primed biopolyester actuators with 50 actuation cycles before force-strain tests to prevent seeing initial behavior.

Specific energies of biodegradable Peano-HASEL actuators in comparison to BOPP actuators force the two different force-strain tests (Fig. 4, C and D) were determined by using the MATLAB function “trapz” and integrating under the respective force-displacement curve. The result was divided by the mass of the tested actuator (without mounts) to calculate specific energy, expressed in joule per kilogram.

Charge leakage during constant DC voltage application (Fig. 4D) was measured by attaching a 10-kilohm resistor in series with the grounding cable connected to an actuator, across which we measured the resulting voltage change. This voltage value (processed using a DAQ) was then converted to current using Ohm’s law: *I* = *V*/*R*. The resistor value was chosen such that the voltage drop would not exceed 5 V, since 10 V is the maximum voltage tolerated by the DAQ. We estimated a maximum current of 0.5 mA, which predicted that 10 kilohms is sufficient resistance.

Displacement of actuators during constant DC voltage application (Fig. 4G) was measured using a laser displacement sensor (Keyence Co., Belgium) aimed from below at the hanging mass (50-g weight) attached to the actuator. Highly dynamic actuations (Fig. 4, H and I) were measured using a high-speed camera (Phantom V2640, USA) with a frame rate of 1000 frames per second and analyzed using Physics Tracker, a free online software for tracking moving objects.

For lifetime tests (Fig. 4H), we created a custom MATLAB program to automatically count the number of cycles of actuation before electrical failure occurred. This was done by forcing the program to stop when a current threshold on the grounded side of the actuator was exceeded because of electrical failure.

Peak strain rate (Fig. 4I) was measured by first using a Savitzky-Golay filter in MATLAB to smooth strain data, then differentiating the resulting strain, and searching for a resulting peak. Ramp time was measured as the time from which voltage is activated to the time that the actuator first reached its equilibrium strain position.

### Fabrication and characterization of a biodegradable SES gripper

Grippers consisted of three sets of two-jointed SES actuators made from BOPLA plastic (fig. S16). Similar to Peano-HASEL actuator fabrication, actuators were heat-sealed and applied with gelatin electrodes 1 mm away from the heat seal edge. Actuators were then attached to a stiff backing made of laser-cut medium fiber density board using a double-sided, acrylate-based adhesive before filling with FR3. A 20-by-20 mm strip of biopolyester film was adhered to the tip of the pincer, onto which talc powder–covered gelatin was applied for use as a compliant end effector. Completed pincers were mounted to a 3D printed base, with each of the high voltage and grounding leads of the three pincers joined together with braided copper tape. The copper was fed to the top of the mount and attached to a portable custom high-voltage driving electronics system (*51*). Applying a voltage using the system caused all three pincers to bend, allowing for object grasping. The electronics and gripper were mounted together onto a robot arm (Panda, Franka Emika Co., Germany).

We characterized the normal force exerted by the tips of individual pincers comprising grippers using previously established methods (*52*); a pincer was exposed to constant DC voltage signals for 4 s, during which normal forces of the pincer tip in relation to distances ranging from 0 to 25 mm from the resting position were measured using a dual-mode lever system (Aurora Scientific 310C-LR, Canada). We attached to the lever system a custom 3D printed lever with an arm length corresponding to the radius of curvature of the pincer tip. Pincers were mounted vertically to an acrylic mount and pressed perpendicular against this custom lever arm (fig. S17). Force measurements were averaged over three cycles of reversing polarity voltage for each input displacement at amplitudes of 4, 5, and 6 kV (fig. S18 and movie S4).

Gripper demonstrations (Fig. 5 and movie S1) consisted of picking up a crumpled paper ball and an empty plastic drinking bottle. We created a preplanned path for the robot arm as well as timed voltage signals for grasping and holding objects.

### Statistics

Unless otherwise stated, data points represent mean values of *n* samples, which are specified in the experiment description, and error bars represent SD.
