# Supplementary material for: Biodegradable electrohydraulic actuators for sustainable soft robots
Source: Sci Adv. 2023 Mar 22;9(12):eadf5551. doi: 10.1126/sciadv.adf5551 (PMC10032599; doi:10.1126/sciadv.adf5551)
Supplement: Supplementary file 1 — Supplementary Text Figs. S1 to S18 Tables S1 and S2 Legends for movies S1 to S4 [file sciadv.adf5551_sm.pdf]

Science Advances
AAAS

Supplementary Materials for

Biodegradable electrohydraulic actuators for sustainable soft robots

Ellen H. Rumley *et al.*

Corresponding author: Christoph Keplinger, ck@is.mpg.de; Martin Kaltenbrunner, martin.kaltenbrunner@jku.at

Sci. Adv. **9**, eadf5551 (2023)
DOI: 10.1126/sciadv.adf5551

The PDF file includes:

Supplementary Text
Figs. S1 to S18
Tables S1 and S2
Legends for movies S1 to S4

Other Supplementary Material for this manuscript includes the following:

Movies S1 to S4

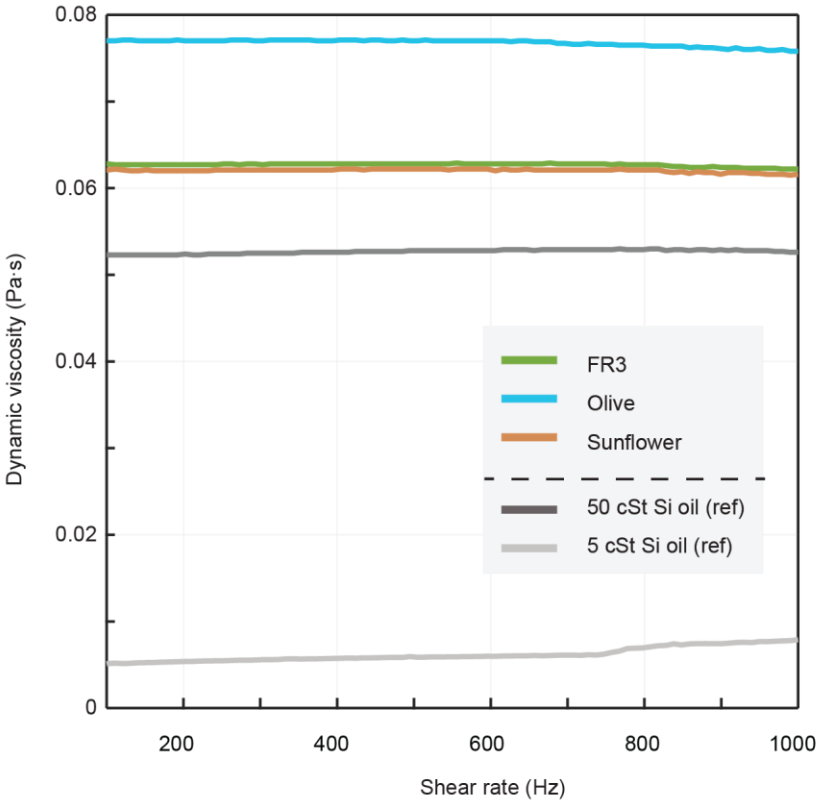

Fig. S1. Dynamic viscosity of biodegradable liquid dielectrics.
FR3, sunflower, and olive oils were measured over a frequency range of 100 to 1000 Hz. Non-biodegradable silicone oils were measured and plotted as reference. Values from the standard frequency of 200 Hz were presented in Fig. 2.

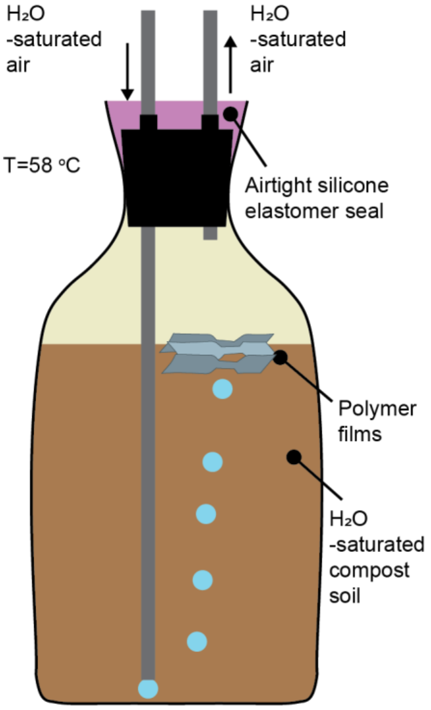

Fig. S2. Schematic of the biodegradation setup.
Dumbbell-shaped BioPolyester samples (same geometry as for tensile tests) were exposed to water-saturated compost soil at a fixed temperature of 58°C in a sealed chamber exposed to humidified air.

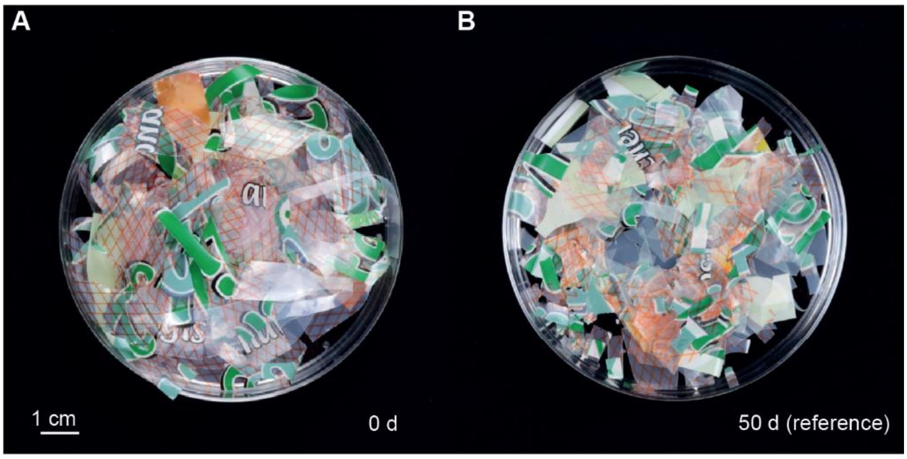

Fig. S3. BioPolyester film in (A) virgin conditions and (B) after submersion in aerated de-ionized water for 50 days at 58°C.

There is no visible difference between the two samples, confirming that films require industrial composting conditions under the presence of microorganisms and enzymes to rapidly biodegrade.

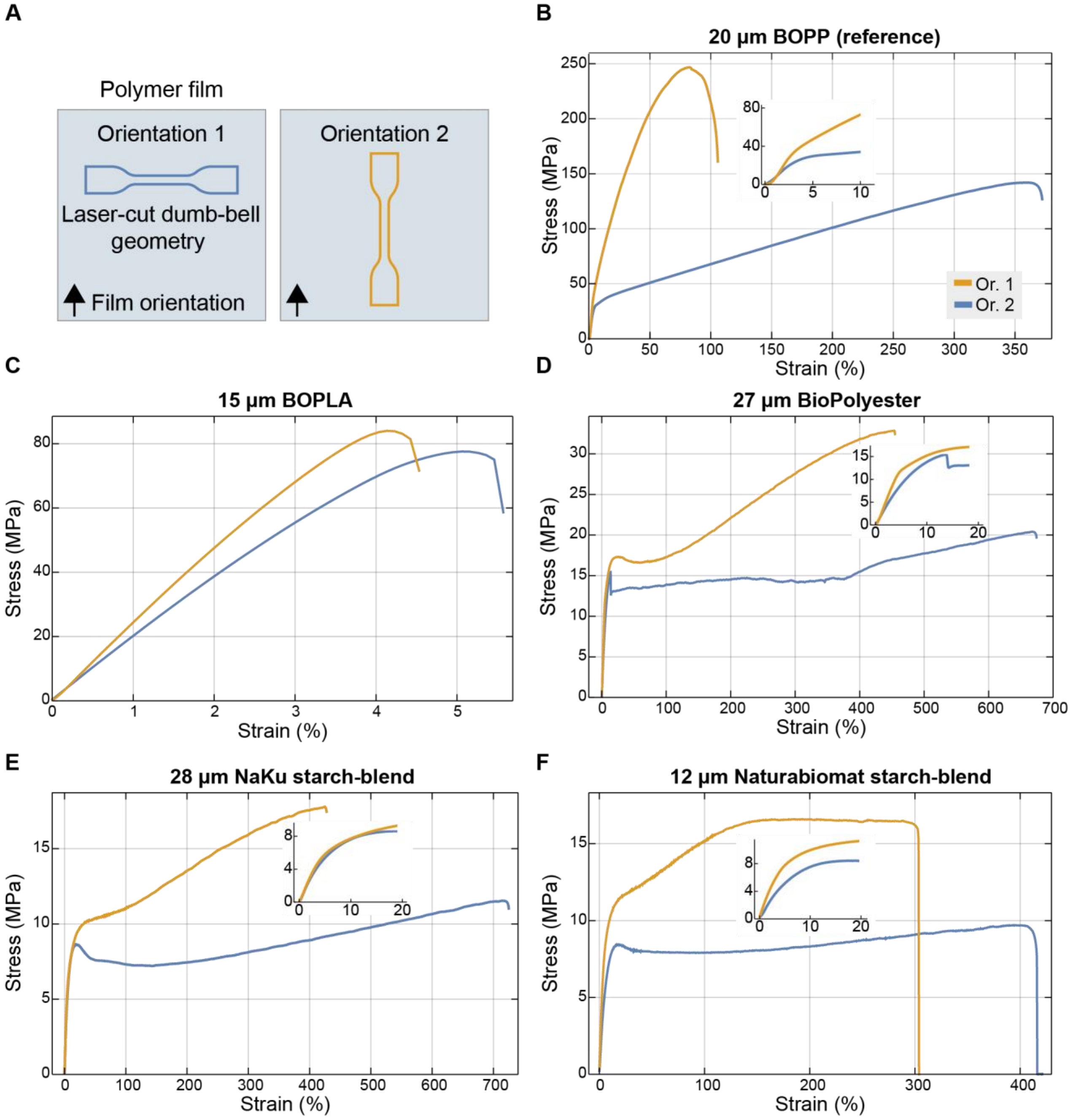

Fig. S4. Uniaxial tensile tests of biodegradable polymer films.

Dumbbell-shaped polymer film samples were laser cut in two orthogonal directions, labeled as “1” and “2”. (Directionality of fabrication was not provided by the manufacturer).

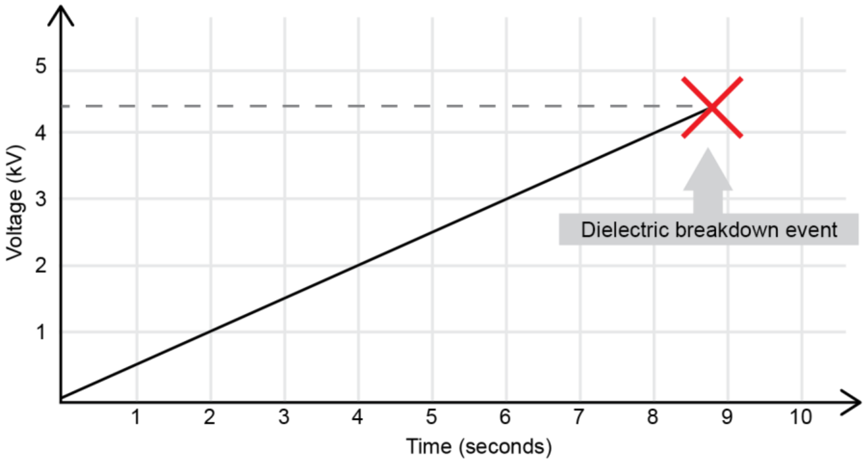

Fig. S5. 500 V/s ramped input voltage signal utilized for testing dielectric strength. A custom LabVIEW program recorded the breakdown voltage.

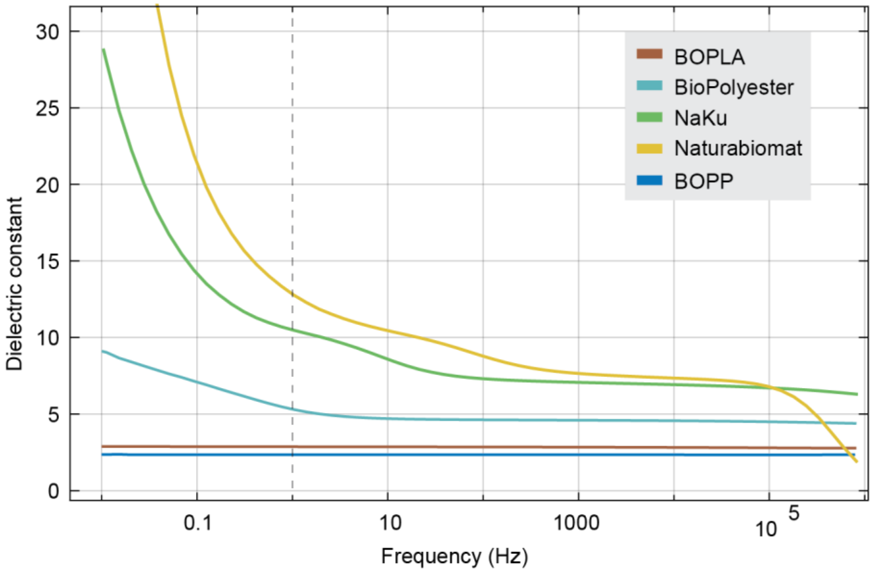

Fig. S6. Dielectric spectra of biodegradable polymer films at V_{AC} = 1 V_{rms}.
The dielectric constants of biodegradable films for calculations of Maxwell stress were taken at a frequency of 1 Hz. BOPP was measured as the reference non-biodegradable film.

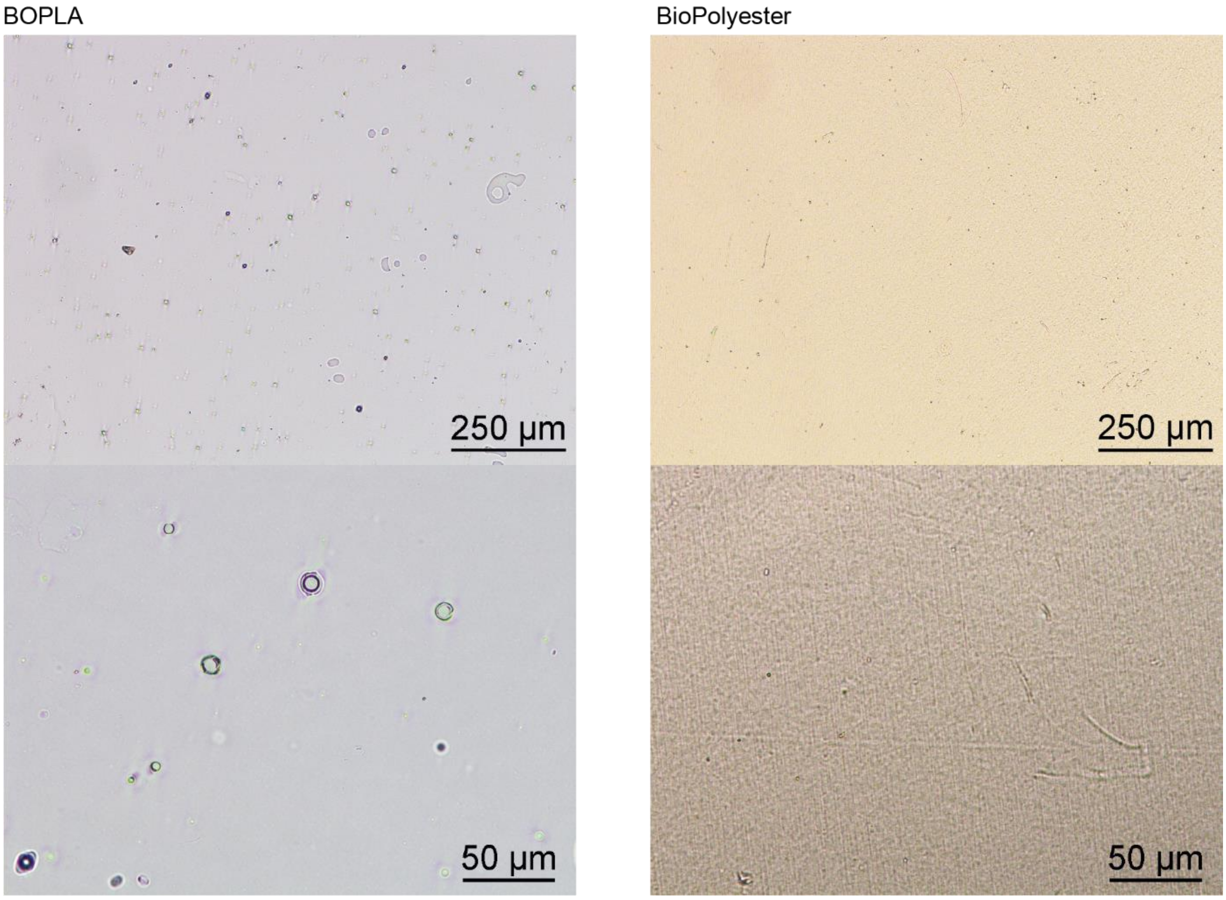

Fig. S7. Optical microscopy images of BOPLA and BioPolyester with (top) 100x and (bottom) 400x magnification.

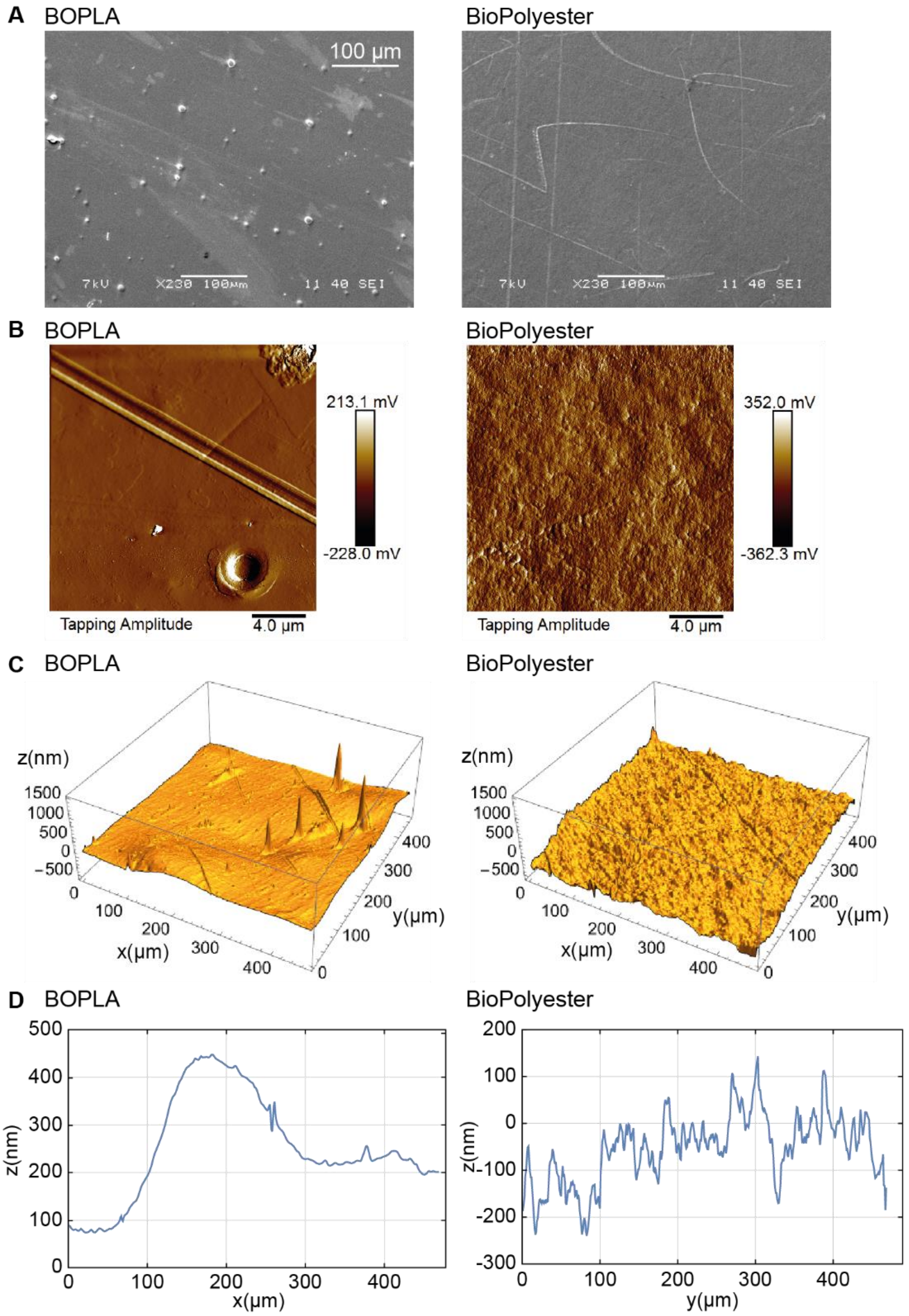

Fig. S8. Surface characterization of BOPLA and BioPolyester.

Surface images of BOPLA (left) and BioPolyester (right) captured by (**A**) SEM at 230x magnification and (**B**) AFM; surface is captured in tapping mode. (**C**) 3D-plots of 0.25 mm^2 surface samples of both biodegradable films obtained with profilometry. (**D**) Height differences of film surfaces visualized using data from profilometry. Data was extracted parallel to y-axis from the plots of (**C**).

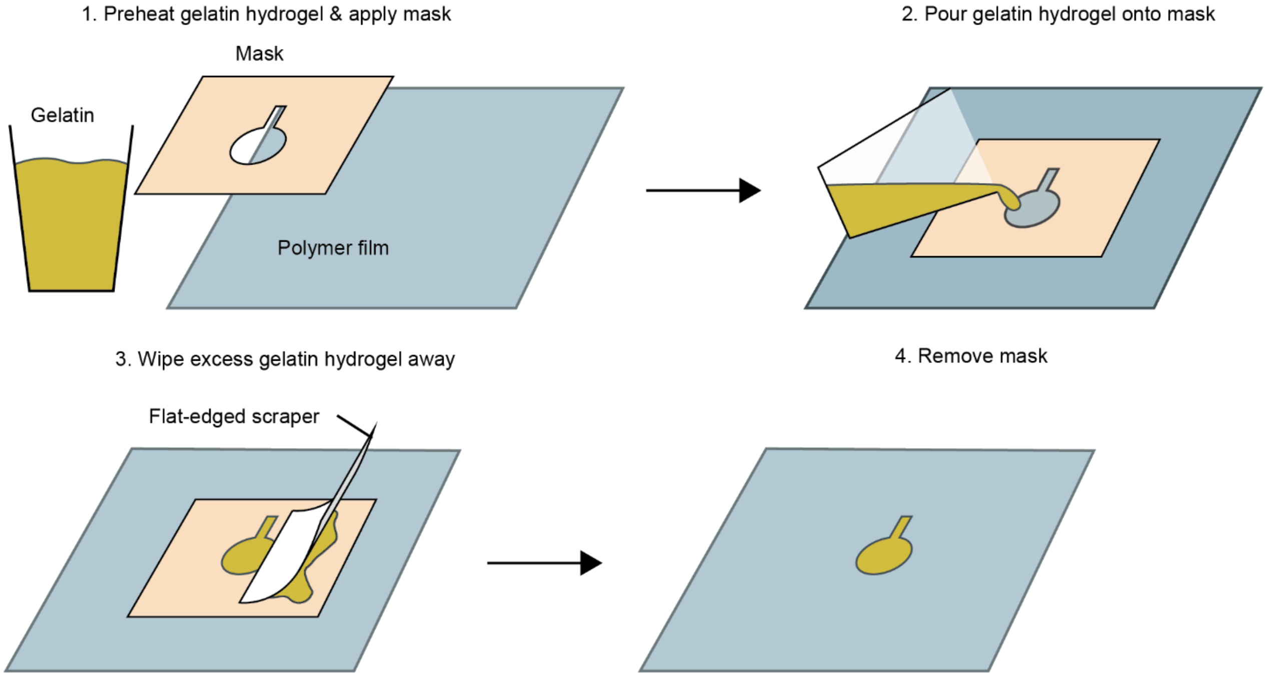

Fig. S9. Fabrication of NaCl-infused gelatin hydrogel electrodes.
Polymer films were taped to a flat surface. An electrode geometry was laser-cut onto a 225-µm thick mask made from screen protector plastic, which was pressed onto the flattened polymer film. Pre-heated gelatin was then cast directly onto the mask. Excess was quickly scraped away with a flat-edged scraper and the mask was promptly removed, allowing the resulting electrode to cure overnight before it was used for testing.

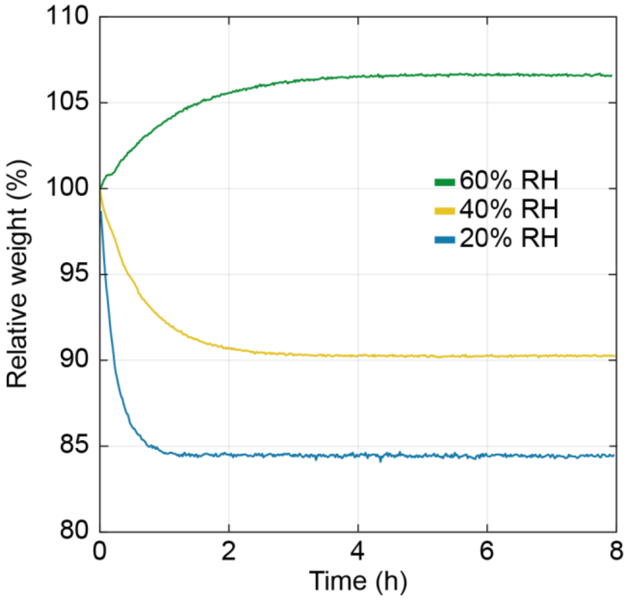

Fig. S10. Relative weight of gelatin hydrogel electrodes at different ambient conditions. Data shows typical weight change, normalized by the initial weight, of gelatin hydrogel stored at 23°C and 20%, 40% and 60% relative humidity over a period of 8 hours.

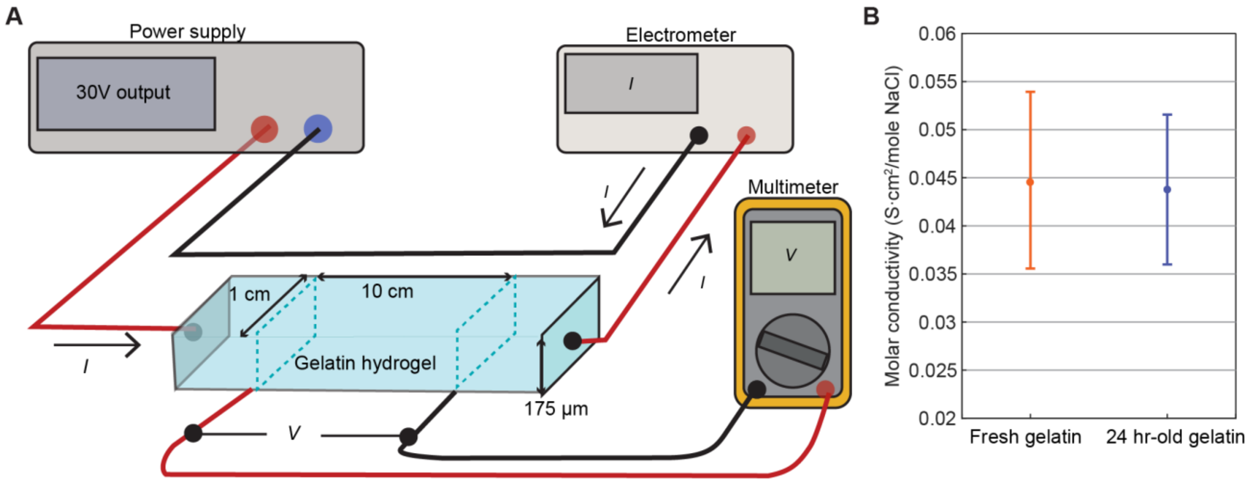

Fig. S11. Conductivity measurement of NaCl-infused gelatin hydrogels.

(**A**) A 4-point measurement setup was used to measure the voltage drop across a known volume of gelatin hydrogel. (**B**) Molar conductivity of gelatin 1) immediately after preparation and 2) after 24 hours of exposure to ambient conditions (23oC, 40% relative humidity). Tests were repeated on 3 samples for each condition.

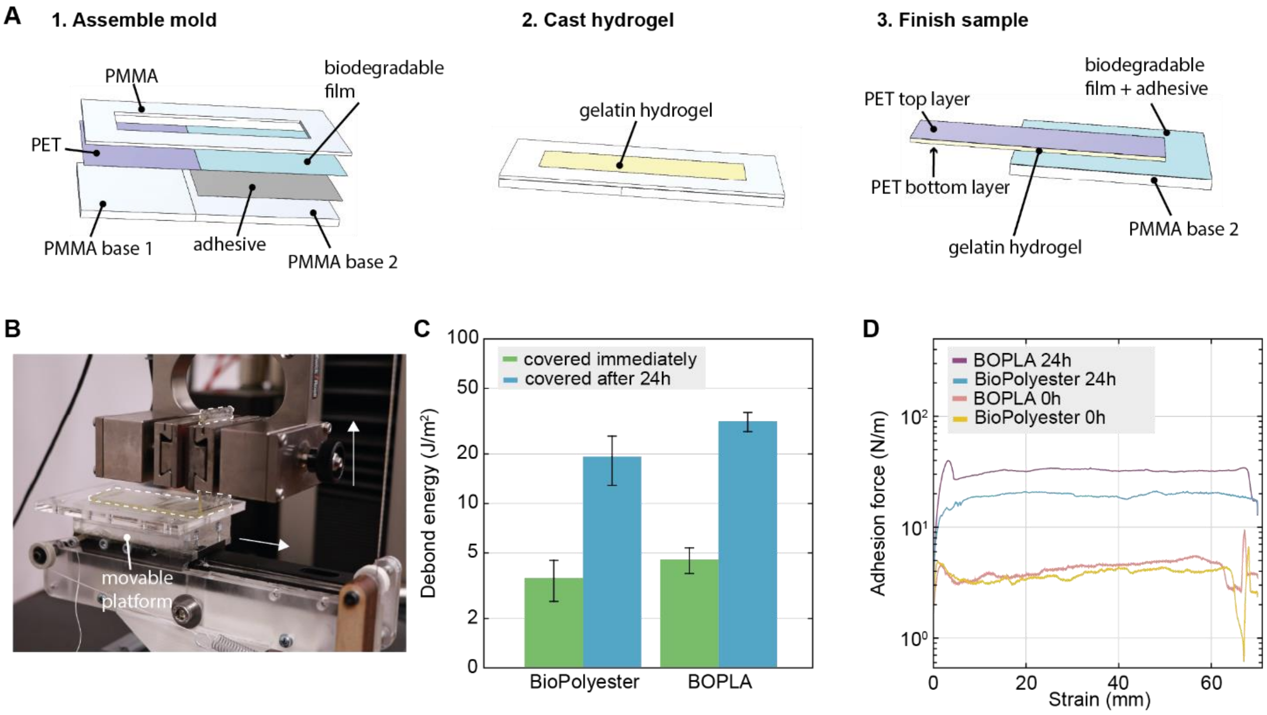

Fig. S12. Peel tests of gelatin hydrogel on biodegradable films.
(**A**) The mold for casting the samples consists of two PMMA base plates, a biodegradable film adhered to the bigger base plate using an intermediate adhesive layer, a layer of PET on top of the smaller base plate and a top PMMA plate. Hydrogel is cast into the mold on top of the films and covered with another layer of PET. All mold parts except for base 2 were removed before testing. (**B**) The peel test setup consists of a movable platform that adjusts according to the travel distance of the clamp. The sample is secured on top of the movable platform and the hydrogel material is put in the clamps in a 90° angle with respect to the biodegradable film. (**C**) Debond energy is calculated from the peeling force of gelatin from biodegradable film samples, for conditions where gelatin is immediately covered with PET after casting or exposed for 24 hours to the ambient environment before PET application. (**D**) Adhesion forces that correspond to data from (B).

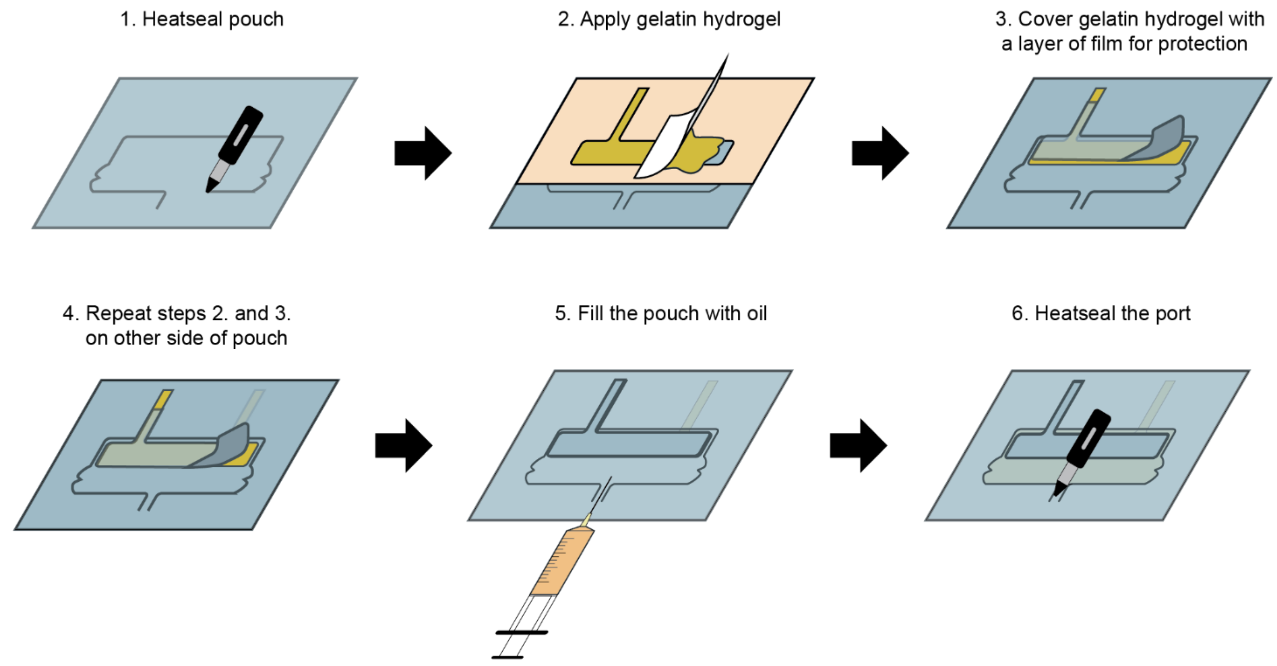

Fig. S13. Fabrication of single-pouch Peano-HASEL actuators.

Two polymer films are heat sealed into a pouch geometry with an opening, using a soldering tip mounted to a pre-programmed CNC bed. Speed and temperature conditions are set for the specific film. Once sealed, gelatin electrodes are applied as depicted in Fig. S9 onto both sides of the actuator, with a sheet of electrode-shaped film laid over the hydrogel as a protective layer. The following day, the pouch is filled with FR3 by inserting a syringe into the pouch opening, which is later sealed off with a soldering iron to complete the fabrication process.

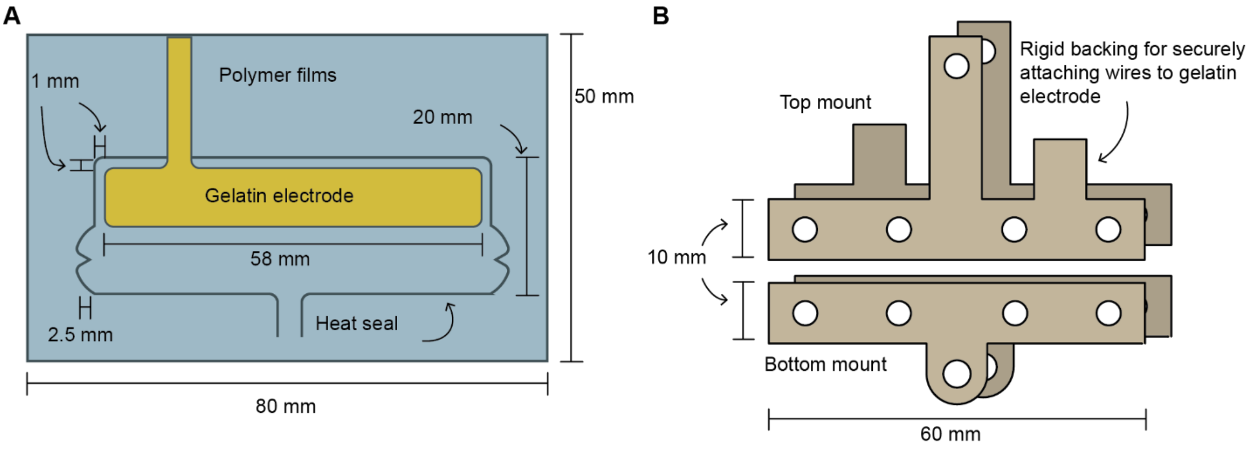

Fig. S14. Geometry of Peano-HASEL actuators.

(A) Peano-HASEL single pouch actuators were heat sealed with 60-mm widths and 20-mm heights. Gelatin hydrogel electrodes had 58-mm widths and 9-mm heights. Ruffles on the bottom half of the heat seal pattern relieved the actuated region of mechanical constraints, which has shown to improve performance in past work. **(B)** Laser-cut 1.5-mm acrylic mounts were mounted 2 mm from the edge of the top and bottom of actuator pouches and secured with screws for actuator characterizations. Hanging masses were loaded onto mounts, which served to distribute load evenly across actuators. A backing was incorporated onto the top mount for securely attaching wires to electrode leads, to avoid ripping hydrogels from the actuator. For Fig. 4, we demonstrated the use of wooden mounts and dowels in substitute of screws.

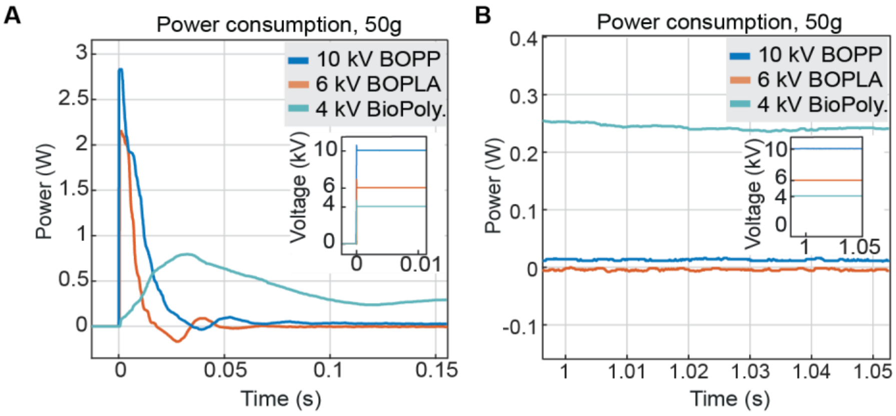

Fig. S15. Power consumption of biodegradable Peano-HASEL actuators under a 50-g load in comparison to non-biodegradable BOPP actuators at (A) instance of voltage application and (B) after one second of constant DC voltage application.

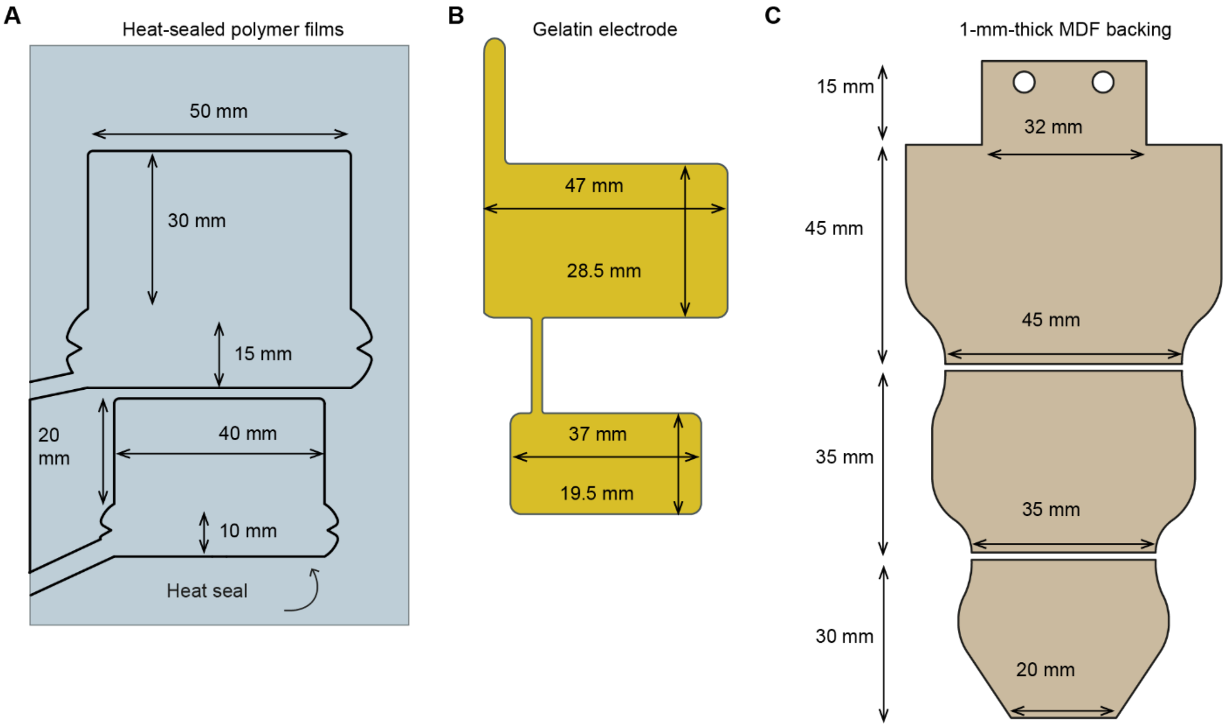

Fig. S16. Biodegradable gripper based on SES joint actuators.

(A) 2-joint SES actuators were sealed from BOPLA film, with a 1-mm gap between each pouch. **(B)** Similar to the Peano-HASEL actuators, gelatin hydrogel electrodes were cast with a 1-mm gap from heat seals. **(C)** SES actuators were attached to a 1-mm thick MDF backing using a double-sided acrylate adhesive. A 20-mm wide strip of plastic was adhered to the bottom tip, lightly coated with gelatin and powdered with talc powder to form a non-sticky compliant end effector.

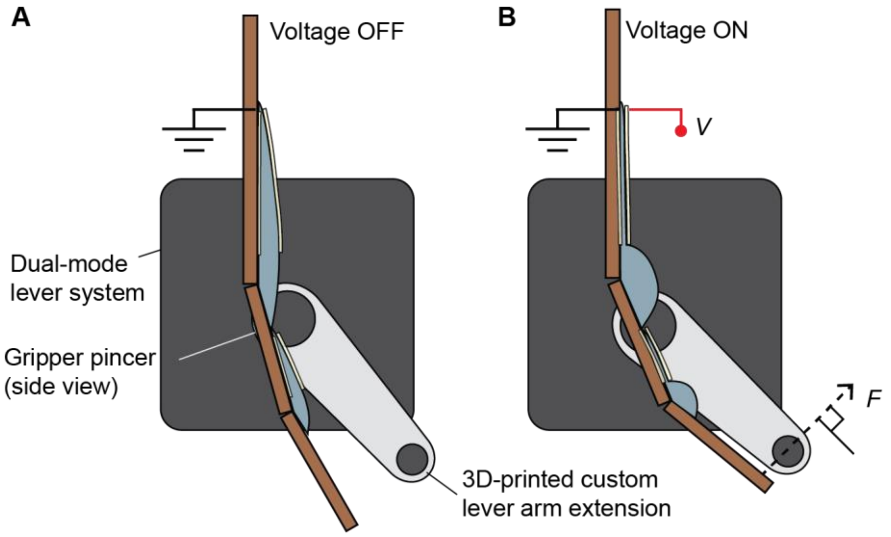

Fig. S17. A dual-mode lever system with a custom 3D-printed lever arm setup used to record the force-displacement of a single BOPLA-based gripper unit (pincer) under different prescibed voltage and displacement conditions.

(A) A pincer in its resting reference position. **(B)** An actuated pincer applies a perpendicular force against the lever arm a fixed distance away.

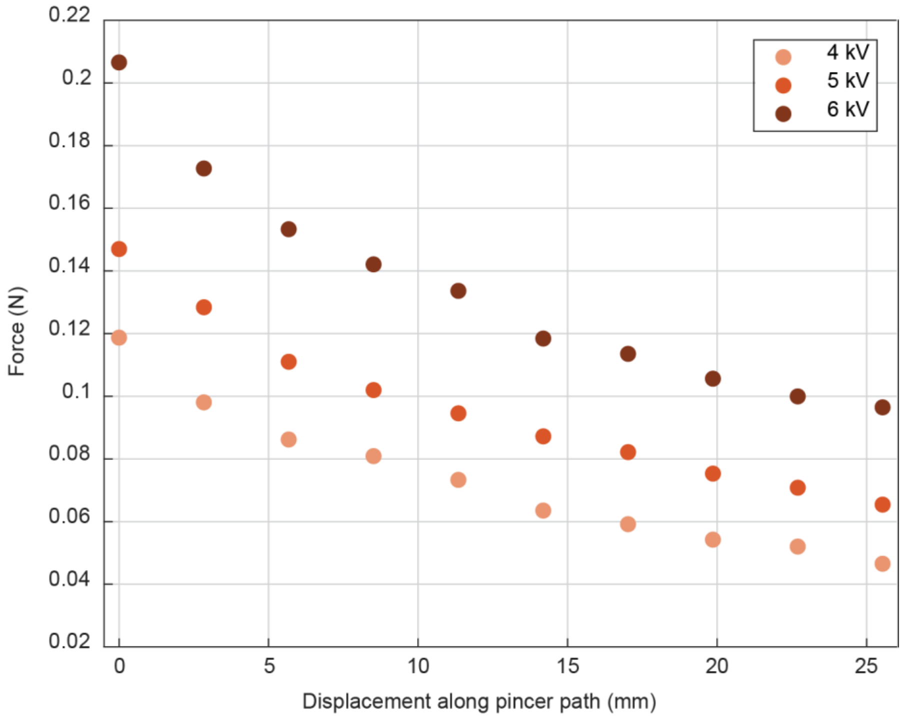

Fig. S18. Voltage-dependent normal force-displacement data of a single BOPLA-based gripper unit (pincer).

Pincers were subject to a voltage signal that ramped up to maximum voltage in 100 ms and held the maximum voltage for 4 seconds before ramping back down and repeating twice more under alternating polarity voltage conditions. Each data point is the result of averaging the force exerted during the three voltage cycles. See Fig. S17 and Movie S4 for visualizing the test setup.

Film	Company	Thickness (µm) n = 15	Breakdown voltage (kV) ASTM D-149 Single film n = 7	Dielectric constant $1V_{rms}$ AC 1Hz n = 2	Dielectric constant $1V_{rms}$ AC 1kHz n = 2	Maxwell stress (MPa) Using dielectric constant@ 1Hz	Young's modulus (MPa) Orientation 1/ Orientation 2 n = 3	Yield strength (MPa) Orientation 1/ Orientation 2 n = 3	Yield strain (%) Orientation 1/ Orientation 2 n = 3
**BOPLA**	**Nativia, distributed from Pütz GmbH + Co. Folien KG**	15.04±0.27	8.39±0.82	2.87±0.00	2.83±0.01	7.9±1.5	20.0±0.12<br>24.9±0.23	52.9±1.00<br>66.4±0.70	2.83±0.06<br>2.85±0.04
**BioPolyester unprinted / (printed)**	**Naturabiomat**	26.5±0.77<br>(26.9±0.72)	5.54±0.15<br>(5.73±0.13)	5.42±0.22	4.73±0.18	2.97±0.1	2.0±0.04<br>2.93±0.09	6.91±0.19<br>10.30±0.50	3.71±0.08<br>3.75±0.08
**Starch-blend "Naturbiomat"**	**Naturobiomat**	12.2±0.63	1.99±0.27	10.46±0.04	7.57±0.08	3.4±0.9	1.30±0.06<br>1.85±0.28	3.57±0.23<br>4.90±0.60	3.07±0.34<br>3.00±0.70
**Starch-blend "NaKu"**	**NaKu**	29.38±2.03	5.37±0.73	12.56±0.22	7.13±0.05	4.4±1.2	1.46±0.22<br>1.81±0.31	3.70±0.50<br>5.00±0.90	3.00±0.80<br>3.10±0.31
**BOPP (reference)**	**Multiplastics Europe Ltd.**	20.31±0.22	13.6±.50	2.19±0.25	2.2±0.02	9±1.9	10.03±1.82<br>16.54±2.96	21.0±4.0<br>33.0±6.0	2.60±0.70<br>2.70±0.90

Table S1.
Standardized measurements of dielectric strengths, dielectric constants, and mechanical properties of four biodegradable films. BOPP serves as a reference non-biodegradable film. "n" denotes the number of independently measured samples.

Film	Weibull cumulative probability of dielectric failure (Fig. 3B) $1 - e^{-(x/\gamma)^{\alpha}}$	Dielectric strength (V/µm) 90% RH ASTM D-149 n = 5	Dielectric strength (V/µm) D = 10 mm copper electrodes n = 5	Dielectric strength (V/µm) D = 10 mm gelatin electrodes n = 5	Dielectric breakdown voltage (kV) 2 films w/ FR3 ASTM D-149 n = 5	Dielectric breakdown voltage (kV) of 2 films heat-sealed along a 80-mm line, parallel to 60-mm-long, 5-mm-wide gelatin electrodes separated by gap **x**. n = 7
**BOPLA**	**For A = 1 cm²** γ^* = 465 $\alpha^\dagger$ = 15 **For A = 10 cm²** γ^* = **405** $\alpha^\dagger$ = **20**	**1 hour:** 565±52 **1 day:** 506±65 **7 days:** 561±60	**0 days:** 499±37 **1 day:** 455±41 **3 days:** 450±21 **5 days:** 481±49 **10 days:** 419±24 **30 days:** 456±51 **50 days:** 464±61	**0 days:** 435±48 **1 day:** 431±25 **3 days:** 421±13 **5 days:** 436±47 **10 days:** 470±20 **30 days:** 462±23 **50 days:** 441±40	**0.1 days:** 13.7±1.20 **1 day:** 15.45±0.89 **3 days:** 14.61±0.93 **10 days:** 14.6±1.80	**x = 0 mm:** 5.11±1.74 **x = 1 mm:** 7.46±1.46
**BioPolyester**	**For A = 1 cm²** γ^* = **197.9** $\alpha^\dagger$ = **12** **For A = 10 cm²** γ^* = **187.3** $\alpha^\dagger$ = **17**	**1 hour:** 223±7 **1 day:** 209±8 **7 days:** 238±11	**0 days:** 218±7 **1 day:** 212.2±3.4 **3 days:** 151±11 **5 days:** 236±14 **10 days:** 206±35 **30 days:** 195±26 **50 days:** 211±12	**0 days:** 159±6 **1 day:** 160±8 **3 days:** 151±11 **5 days:** 163±8 **10 days:** 182±15 **30 days:** 152±25 **50 days:** 148±6	**0.1 days:** 7.18±0.14 **1 day:** 6.90±0.12 **3 days:** 7.59±0.34 **10 days:**7.50±0.27	**x = 0 mm:** 5.70±0.76 **x = 1 mm:** 6.51±0.66

Table S2.

Environment-dependent dielectric strength of BOPLA and BioPolyester films. "n" denotes the number of independently measured samples. † = shape parameter, ***** = characteristic breakdown strength of the material, taken as the breakdown field where 63% of polymer samples experienced failure**.**

Movie S1.
Robustness of adhesion between gelatin hydrogel and biodegradable polymer films under repetitive bending stress.

Movie S2.
"Wobbling" behavior of BioPolyester Peano-HASEL actuators.

Movie S3.
Demonstration of a biodegradable SES gripper.

Movie S4.
Setup for measuring the normal force exerted by pincer tips comprising biodegradable SES grippers.
